# Knowledge and practices of traditional management of child malnutrition and associated pathologies in Benin

**DOI:** 10.1186/s13002-024-00684-x

**Published:** 2024-05-02

**Authors:** Ahamidé Carel Sosthène Vissoh, Jean Robert Klotoé, Lauris Fah, Eric Agbodjento, Hornel Koudokpon, Eskyl Togbe, Souad Saïdou, Victorien Dougnon

**Affiliations:** 1 Research Unit in Applied Microbiology and Pharmacology of natural substances, Polytechnic School of Abomey-Calavi, University of Abomey-Calavi, Abomey-Calavi, Benin; 2 Multidisciplinary Research Laboratory for Technical Education (LARPET) of the National Higher School for Technical Education (ENSET) of Lokossa, National University of Science, Technology, Engineering and Mathematics (UNSTIM), Abomey, Benin; 3 National Medico-Sanitary Institute (INMeS), University of Abomey-Calavi, Abomey-Calavi, Benin

**Keywords:** Child malnutrition, Associated pathologies, Medicinal plants, Benin, Traditional healers, Mothers

## Abstract

**Background:**

Child malnutrition is a major public health challenge, affecting millions of children worldwide, with alarming proportions of children under five in Benin. The complexity of managing this condition is increased by its potential association with opportunistic pathologies. An interesting approach arises from the use of medicinal plants, to address child malnutrition and its associated pathologies. This study aimed to document the knowledge and practices of Beninese mothers and traditional practitioners with regard to the use of medicinal plants to treat child malnutrition and associated diseases.

**Methods:**

A total of 844 Beninese mothers and 201 traditional healers were surveyed between March 2022 and August 2023 in the communes of Karimama, Bopa and Za-Kpota in Benin. The respondents’ knowledge of child malnutrition and associated pathologies was explored. The ethnobotanical data collected from the subjects concerned the medicinal recipes used to treat child malnutrition, the medicinal plants that make them up and the methods of use. These data were analyzed using ethnobotanical indices such as the Informant Consensus Factor, the frequency of citation of medicinal recipes types and medicinal plants, and the contribution of plants to medicinal recipes.

**Results:**

All respondents cited a total of 82 plant species used to treat child malnutrition and associated diseases. These plants were grouped into 37 botanical families, the most common of which were Fabaceae, Malvaceae and Annonaceae. The leaves were the most commonly used part of the plant species identified. The mothers shared 122 medicinal recipes, ranging from recipes based on a single plant to more complex compositions involving five plants. The most notable plants were *Moringa oleifera* Lam, *Phyllanthus amarus* Schumach & Thonn, *Senna siamea* (Lam.) H.S.Irwin & Barneby, *Carica papaya* L. and *Ocimum gratissimum* L. Traditional healers provided 52 plants in 71 recipes, with *Moringa oleifera* featuring prominently in both single-plant and multiplant formulations.

**Conclusion:**

This study made it possible to constitute a rich base of medicinal recipes used against malnutrition and associated pathologies, with the preponderant involvement of certain plant species. It is therefore necessary to deepen research on these different identified species in order to scientifically assess their potential.

**Supplementary Information:**

The online version contains supplementary material available at 10.1186/s13002-024-00684-x.

## Background

Child malnutrition remains a major challenge for public health, affecting nearly 207 million children worldwide and contributing to about 45% of deaths among children under 5 years old [1–3]. According to the WHO, malnutrition in an individual is the imbalance between the nutrients received through diet and their energy needs [4]. Underlying factors for malnutrition include poverty, lack of access to quality food, unhealthy environments and inadequate care during the first two years of life [5].

Globally, three main regions (South Asia, sub-Saharan Africa and Latin America) concentrate the majority of cases of child malnutrition, leading to severe acute malnutrition responsible for 4.4% of deaths in children under 5 years old [6, 7]. This form of malnutrition is associated with complications such as diarrhea, measles, acute respiratory infections, anemia, malaria and HIV/AIDS (human immunodeficiency virus/acquired immunodeficiency syndrome) [8, 9].

In Benin, malnutrition persists with significant disparities between regions and municipalities. In 2018, in the commune of Karimama, 30.8% of children under 5 were underweight, 13.0% were emaciated and 40.3% were stunted [10]. Similar figures are observed in the Za-Kpota municipality, Zou department, with a prevalence of 32.0% stunted growth, 25.8% underweight and 5.2% severe wasting in children under 5 years old [11]. These alarming rates of chronic malnutrition, underweight and stunted growth emphasize the need for innovative and accessible solutions to improve the management of these health issues. The World Health Organization (WHO) highlights that efficient management of malnutrition must take into account pathologies associated with child malnutrition. It recognizes the relevance of this therapeutic approach and has recommended combining substances with antibiotic properties with those possessing nutritional potential for effective management of severe acute malnutrition [12]. Traditional management of severe acute malnutrition, in line with WHO guidelines, involves the administration of therapeutic ingredients and foods. However, challenges such as high costs, logistics and dependence on these products underscore the need for more holistic and sustainable approaches in developing countries [13]. A promising alternative is community-based management recommended by the WHO, emphasizing the use of local preparations to treat malnutrition [4]. Studies in Benin have demonstrated the effectiveness of local food formulations [14–17], but challenges persist in terms of accessibility and treating associated pathologies.

In the Beninese context, mothers and traditional healers play crucial roles in the care and well-being of children, particularly in terms of health [15, 18]. Mothers take on the primary role as caregivers, being responsible for the daily health and nutritional needs of their children. On the other hand, traditional healers are influential figures equipped with specialized knowledge in medicinal plants and traditional healing practices. The combination of maternal perspectives and the expertise of traditional healers could provide a comprehensive understanding of local knowledge and practices related to child malnutrition and its associated pathologies. With their specialized knowledge in traditional medicine, traditional healers could offer unique perspectives, knowledge and practices influenced by their respective roles and experiences. They could provide information on specific medicinal plants, formulations and traditional treatment methods. Mothers, on the other hand, being in direct and constant contact with their children, could shed light on daily challenges, nutritional practices and home remedies. Furthermore, several variables such as age, religion and marital status can influence the use of plants based on the cultural context and practices specific to each community in the context of traditional management of child malnutrition and associated pathologies in Benin. Regarding age, generational differences can influence knowledge and preferences in traditional medicine. Older generations may possess deeper knowledge of medicinal plants, while younger individuals might be more open to modern medical practices. Marital status can influence family responsibilities and, consequently, knowledge in health care. Mothers, for example, might be more involved in the use of plants for the well-being of their children compared to other groups. Religious beliefs can also play a role in the choice of medicinal plants. Some religious groups may have specific preferences or restrictions regarding medical treatment, which could influence the reliance on traditional plants.

In Benin's long tradition, traditional medicine based on the use of medicinal plants plays an important role in the therapeutic arsenal of over 80% of the population [19, 20]. These plants, in addition to their nutritional potential, possess antibiotic properties that could be useful in managing child malnutrition and associated pathologies. In Benin, despite numerous studies on malnutrition management through various processes, it is evident that the proposed local treatments or formulations only aim to improve the nutritional status of malnourished children [14–17]. For instance, the study by Bodjrenou et al. [14] demonstrated the effect of an improved traditional porridge, prepared from a blend of maize, groundnut, cowpea and malt, on the nutritional status of children aged 6–12 months in a rural area of southern Benin. The results highlighted that the intervention led to improved energy, protein, vitamin A and iron intake among children who received the enriched flour-based porridge. However, it did not address other aspects of the problem. Fanou-fogny and et al. [15] investigated the formulation of fonio flour enriched with local food resources for the complementary feeding of young children in Benin. The results showed that Ffo2 fonio porridge contributes to the minimum dietary diversity of children aged 6–23 months, by combining at least four of the seven recommended food groups: cereals (fonio), legumes (soya), seeds (groundnuts) and fish (fry).

The flora of Benin is rich and diverse, comprising between 2500 and 3000 plant species. It presents more than 2,800 taxa, with information on the type of environment in which they live (semi-deciduous dense rainforest, grassy savannah, wooded savannah, mangrove, etc.) and the names of a few localities where they have been observed or collected. The book is highly recommended for traditional practitioners in Benin and also provides plant names in 21 national languages in addition to French [21]. Based on the floristic diversity of Benin, it is possible to provide a local solution using medicinal plants for the traditional management of child malnutrition and associated pathologies.

Exploratory research on the knowledge and practices of traditional medicine practitioners and mother of children in Benin is necessary to understand how traditional management of malnutrition and associated pathologies can be improved in an integrated manner. This study aimed to document the knowledge and practices of Beninese mothers and traditional healers regarding the traditional management of child malnutrition and associated pathologies.

The hypotheses of this study are:

- The utilization of medicinal plants in traditional medicine in Benin significantly contributes to the improvement of child malnutrition and associated pathologies.
- Traditional medicine practitioners in Benin possess valuable knowledge and practices that can enhance the traditional management of malnutrition and associated pathologies

## Materials and methods

### Study area

**Fig. 1:**
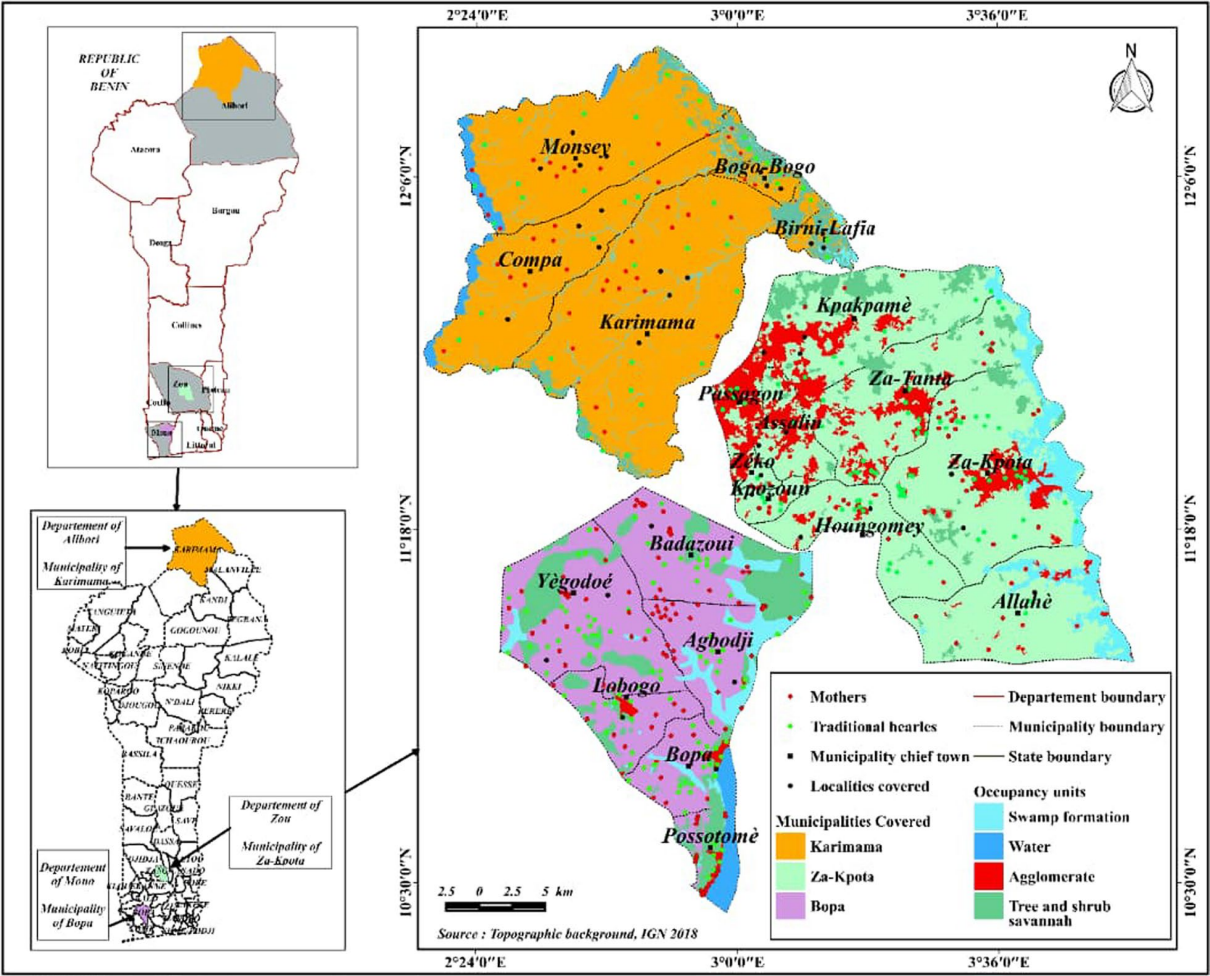
Map showing the surveyed communes of Za-Kpota, Bopa and Karimama

This study was conducted in three municipalities in Benin (Fig. 1): Karimama in the north, Za-Kpota in the center and Bopa in the south (Table 1). These three municipalities were selected using a reasoned choice methodology. To allow for longitudinal investigations of the issues addressed in this study, the three main regions, namely north, center and south Benin, were considered. In each region, the municipality with a high prevalence of acute malnutrition was identified from the health zones according to the MICS (Multiple Indicator Cluster Surveys) 2014 study [22]. Karimama is a municipality and city located in the northeast of Benin, in the Alibori department. Bordering Niger, it is the northernmost municipality in the country. It has a Sahelo-Sudanese climate with two seasons: the dry season extending from November to mid-May and the rainy season from mid-May to October. Average annual precipitation is about 600 mm. Average temperatures are around 40 °C in April–May–June, but range from 12 to 25 °C from November to March during the harmattan. The municipality has 66,353 inhabitants, including 11,901 in the Karimama district. Bopa, with an area of 365 km^2^, constitutes 22.7% of the Mono department's area and has approximately 96,281 inhabitants. It is a commune in the southwest of Benin, the capital of the Mono department. It is characterized by a subequatorial climate with four seasons: a large dry season (November to March), a large rainy season (March to July), a small dry season (July to August) and a small rainy season (August to November). Annual average rainfall ranges between 800 and 1000 mm, making the climate favorable for mixed farming. The vegetation is characterized by forest species such as *Adansonia digitata* L. (baobab), *Rhodognaphalon brevicuspe* (Sprague) Roberty (silk-cotton tree), kapok tree and *Azadirachta indica* A.Juss. (neem). Za-Kpota is located in the Zou department. The population of Za-Kpota was 132,818, including 61,945 men and 70,873 women (RGPH4) (General Population and Housing Census). The commune covers an area of 409 km^2^. The dominant sociocultural group is Fon (90%). Traditional religion is the most dominant in the commune (75%), followed by Christianity (24%) and Islam (1%) [23].

**Table 1:** Demographic, ethnic, linguistic, religious and ecological characteristics of three municipalities in Benin: Karimama, Za-Kpota and Bopa

Communes	Karimama	Za-Kpota	Bopa
Ethnicity	Dendi, peulh, gourmantché, Haoussa, Yorouba, Fon, Bariba	Fon, Mahi, Yoruba, Dendi	Sahouê, les Ayizɔ, les Péɖah, les kɔtafɔn, Fɔn, les Mina, les Adja
Local language	Dendi	Fon	Sahouê
Religion	Muslim (91.3%); Traditional; Catholic (1.5%); Protestant (0.1%), Other (3.5%)	Traditional (75.4%), Catholic (7.7%), Protestant (1.1%), Muslim (0.5%), Other (15.3%)	Traditional (83.1%); Muslim (0.3%), Catholic (11.2%), Protestant (0.6%), Other (4.8%)
Number of inhabitants	People: 66.353	People: 132.818	People*:* 96 281
Ecology (climate and vegetation)	Karimama enjoys a Sahelo-Sudanese climate with two distinct seasons: The dry season, from November to mid-May. The rainy season, from mid-May to October. Average annual rainfall is around 600 mm The average temperature varies from 40 °C during April, May and June, to 12 to 25 °C from November to March, when the harmattan blows. Karimama's vegetation consists mainly of Sudanian and Sudano-Sahelian savannah There are also gallery forests along the watercourses Some of the plant species found in the region	The commune of Za-Kpota has a tropical climate with two seasons: Rainy season: This generally runs from March to November, with heavier rainfall during this period Dry season: The dry season runs from December to February, with warm temperatures but less rainfall. Due to the tropical climate, the dominant vegetation in the commune of Za-Kpota is dense tropical forest. There are areas of savannah in the surrounding area, characterized by tall grasses and scattered shrubs	Bopa is a commune in Benin with a characteristic dry winter savannah climate and two main climatic seasons: Dry season (November to March and July to September): During these periods, rainfall is less frequent The climate is drier, with high temperatures Wet season (April to July and September to mid-November): During these periods, rainfall is more abundant The vegetation is more lush, and temperatures can be more moderate. Bopa's vegetation consists mainly of Sudanian and Sudano-Sahelian savannah. There are also gallery forests along the watercourses

### Targets and sample size

The survey targeted mothers of children and traditional healers (Table 2). The data for this study were collected from March 2022 to August 2023.

**Table 2:** Occupations and demographic characteristics of study participants

Study participants	Number	Genders	Age group (Years)	Occupation
Traditional healers	76	Female	21–60	Herbalist
	78	Men	21–60	Traditherapist
	52	Men	21–60	Phytotherapist
Mothers of children	610	Female	10–60	Housekeeper
	193	Female	10–50	Clerk
	19	Female	10–40	Civil servants
	23	Female	10–50	Other

#### Mothers of children aged under five

The recruitment of mothers of children aged under five for the study was conducted with a strong emphasis on ethical principles, transparency and inclusivity, ensuring that the sample represented the diverse population under investigation. Community engagement played a crucial role in the recruitment process. Close collaboration with local communities was established, working hand in hand with local authorities, community leaders and health centers. This collaborative approach facilitated the seamless integration of the study into the local context, building trust and fostering cooperation among the participating mothers. Prior to their participation, mothers were provided with comprehensive information about the study's objectives, the nature of their involvement and the ethical considerations involved. Informed consent procedures were meticulously implemented, allowing mothers the opportunity to ask questions and voice any concerns before agreeing to take part in the survey. Inclusivity was a guiding principle throughout the recruitment process. Researchers made concerted efforts to include mothers from diverse geographical regions and various socioeconomic backgrounds. This approach ensured a balanced representation of diversity within the sample, thereby enhancing the validity of the study results by capturing the different realities experienced by mothers across Benin. As the study was cross-sectional and analytical, the Schawrtz formula was used to determine the size of the study population for mothers looking after children under 5 [24]:

$$N=\frac{{ \mathrm{Z\alpha }}^{2}pq}{{i}^{2}}$$

*α*: is the accepted risk of error = 5%

*Zα*^2^ = 1.962 (reduced deviation corresponding to the risk of error *α* at 5%).

*p*: the prevalence of knowledge of malnutrition among mothers of children under 5. In the absence of previous studies, *P* = 50.00% was used.

$$q\, = \,1 - p\, = \,1 - \, 0.50$$

*i*: desired accuracy = 5%.

In accordance with the WHO cluster sampling method [25], the cluster effect was applied to the study population to obtain the final sample size of 844 people.

#### Traditional healers

Regarding traditional healers and herbalists, a random sampling coupled with the snowball sampling method was employed [26].

The random selection of traditional healers was executed systematically to ensure a representative sample for the study. First, a directory of local traditional healers was compiled using community and regional sources, establishing a comprehensive list of practitioners in the area. From this directory, a random sampling method was applied with the collaboration of village chiefs. This approach helped identify renowned traditional practitioners with significant experience in traditional medicine, ensuring a diverse representation of expertise within the sample. Inclusiveness was a key principle guiding the selection process. Traditional healers from various geographical zones and medicinal traditions were deliberately included, aiming for a broad spectrum of practices to be observed and analyzed. This approach enriched the study by capturing the nuances and variations present in traditional healing practices across different cultural and geographical contexts. Furthermore, ethical considerations were prioritized throughout the selection process. Traditional healers were fully informed about the study's objectives, methodologies and potential implications. They were also provided with detailed information about their rights as participants and the confidentiality of their responses. Informed consent was obtained from each traditional healer before their involvement in the study, ensuring their voluntary and informed participation in the research endeavor. This adherence to ethical standards underscored the commitment to conducting the study with integrity and respect for the rights and dignity of all involved parties. In total, 201 traditional healers and herbalists were included in this study.

### Data collection

The principles of the code of ethics of the International Society of Ethnobiology relating to this study were observed during data collection [27]. A consent form for participation and data protection was signed by the participants before this study (https://drive.google.com/file/d/18DAihEGj8ZXBWNPC5sb5bXaLoHPrazzm/view?usp=sharing).

#### Data collection on respondents’ knowledge of malnutrition

An investigation was conducted using the semi-structured interview method, targeting mothers caring for children under 5 years old and traditional healers and herbalists in the markets of these municipalities [28]. The questionnaire was designed via KoboCollect for each target group, and the free and informed consent of each respondent was obtained in writing before its administration.

The targets were surveyed in Birni Lafia, Karigui, Bobo-Bobo, Kompa, Kompanti, Petchinga and Karimama I for the Karimama municipality. Those from the Bopa municipality were surveyed in the villages of Agbodji, Koweho, Medetogbo, Missiafo, Zoungbo, Kpave, Djekian, Tekozouin, Akokponawa. In the Za-Kpota municipality, the survey was conducted in the villages of Kpindjigbedji, Dogbanlin, Assanlin, Aketekpa, Koguede, Kpakpame, Ahossougon, Kpozoun Zoungoudo, Adikogon, Agonkanme, Tanta, Zeko, Djoyitin, Sohounta (Fig. 1). Interviews were conducted in one of the local languages (Fon, Goun, Mahi, Aïzo and Adja).

The socio-demographic data collected of the surveyed include the occupation of the respondents, the age distribution (stratified into different brackets, ranging from 10 to 20 years, 21 to 30 years, 31 to 40 years, 41 to 50 years and 51 to 60 years), participants’ marital status (“Single,” “Divorced,” “Married” and “Widowed” categories), level of education (“Illiterate,” “Primary,” “Secondary” and “University”), source of endogenous medicinal knowledge and finally, ethnicity (Adja and related, Fon and related, Dendi and related, Gourmantché, Peulh and related, Yoa and related, and Yoruba and related).

The knowledge of the subjects interviewed about child malnutrition was assessed through the definition of child malnutrition, its causes, symptoms and consequences, as well as the most frequently associated pathologies.

#### Collection of ethnopharmacological data

The ethnopharmacological data collected covered traditional plant-based remedies, the vernacular names of the plants, the plant parts used, their state of use, the criteria for using the recipe, the specific treatment targeted by each medicinal recipe, the dosage, the method of preparation and the route of administration [28].

#### Identification of plants

The plants collected were identified and certified at the Benin National Herbarium using the analytical flora of Akoègninou et al. [21] by Professor YEDOMONHAN Hounnankpon, curator of the Benin National Herbarium. Herbariums were compiled and deposited at the National Herbarium of Benin at the University of Abomey-Calavi. The botanical nomenclature of Plants of the World Online website (https://powo.science.kew.org/) was used to confirm the complete names of all medicinal plants listed in this study.

#### Data processing

The recorded data on the survey forms were then entered into the Excel 2016 spreadsheet and subjected to statistical analysis using SPSS 26.0 software. Socio-demographic data were analyzed using descriptive statistics (total and percentage). Regarding the evaluation of respondents' knowledge of infant malnutrition, given that the level of knowledge varies from one individual to another, a classification into three distinct categories was performed: low knowledge, average knowledge and good knowledge based on the FAO (Food and Agriculture Organization) guide [29] and WHO guidelines [12]. Indicators for quantifying knowledge were defined as percentages for these three categories in accordance with the WHO guidelines Indicators for quantifying knowledge were defined as percentages for these three categories in accordance with the WHO guidelines [12]. In additional, five ethnobotanical indices were used to analyze the ethnopharmacological data collected:

- Frequency of medicinal plant citations (*F*c)

The frequency of medicinal plant citations (*F*c) used to identify the most plants used by the respondents for the traditional treatment of malnutrition and pathologies associated. This was determined using the following formula [26]:

$${\text{Fc}} = \frac{{{\text{number}}\; {\text{of}}\; {\text{citations}} \;{\text{of}} \;{\text{species}}}}{{{\text{total}} \;{\text{number}} \;{\text{of}}\; {\text{citations}} \;{\text{for}}\; {\text{all}} \;{\text{species}}}}*100$$

- Frequency with which medicinal recipes (Fcr) are cited

The recipes with the highest Fcr values reflect the recipes most used by respondents. This frequency was calculated using the formula applied by Dassou *et al.* [30]

$${\text{Fcr}} = \frac{{{\text{number}}\; {\text{of}} \;{\text{citations}} \;{\text{of}}\; {\text{a}}\; {\text{typical}}\; {\text{medicinal}}\; {\text{recipe}}}}{{{\text{total}}\; {\text{number}}\; {\text{of}}\; {\text{all}} \;{\text{citations}} \;{\text{of}}\; {\text{this}} \;{\text{type}}\; {\text{of}}\;{\text{ medicinal}}\; {\text{recipe}}}}*100$$

- Informant consensus factor (ICF)

The Informant Consensus Factor (ICF) serves as a crucial tool in evaluating the coherence of information provided by respondents [31, 32]. Within the framework of this study, the ICF was calculated based on informant categories using the prescribed formula: ICF = (Nuc − Ns) / (Nuc − 1) [30]. Here, Nuc represents the number of use citations within each informant category, while Ns signifies the number of plant species cited by informants in that particular category. The ICF ranges from 0 to 1, where a value of 0 indicates the lowest degree of consensus, reflecting diverse perspectives among respondents regarding the use of plants for treating the illness. Values below 0.5 are considered weak, denoting a low consensus. A value of 0.5 reflects the average degree, indicating a moderate consensus, while values between 0.5 and 1 represent strong degrees, showcasing a relatively high agreement in the use of plants for treatment. The highest degree, a value of 1, signifies complete consensus in the use of the identified plants for treating the illness.

The contribution of each plant species to recipe (Cpr) was determined using the formula described by several authors [30, 33, 34]:

$$Cpr \, = \, \left( {N{\text{r }}/ \, N{\text{t}}} \right) \, \times \, 100$$

*N*r: number of recipes involving the plant species and *N*t: total number of recipes.

- Fidelity index

The fidelity index (FI) helps to assess the intensity of the relationship that informants establish between a medicinal plant and its role in treating a disease or a category of diseases. This index is based on the percentage of informants who confirmed the use of a plant in the treatment of the targeted disease.

The fidelity index (FI) is used to assess the strength of the relationship that informants establish between a medicinal plant and its role in the treatment of a disease or category of diseases. This index was used to identify the plants most commonly used by respondents to treat malnutrition and related illnesses.

It is calculated using the following formula [35]:

$${\text{IF}}=\frac{I{\text{p}}}{I{\text{u}}}*100$$

*I*p is the number of informants who have used the given species to treat a specific category of diseases and *I*u is the total number of surveyed informants. In this study, this index was determined by category of informants.

## Results

### Socio-demographic characteristics of respondents

In total, 844 mothers of children were surveyed. These included homemakers, traders and civil servants. Predominantly married, illiterate and belonging to the Fon and related ethnic groups, these women were in the age group between 21 and 40 years (Table 3).

**Table 3:** Socio-demographic of surveyed mothers

Variables	Categories	Total	Percentage (%)
Occupation	Housekeeper	593	71.77
	Shopkeeper	209	23.51
	Civil servant	19	2.14
	Other	23	2.59
Age	10–20 years old	30	3.37
	21–30 years old	403	45.33
	31–40 years old	374	42.07
	41–50 years	67	7.54
	51–60 years old	15	1.69
Marital status	Single	35	3.94
	Divorced	11	1.24
	Married	815	91.68
	Widowed	28	3.15
Education level	Illiterate	541	60.85
	Primary	231	25.98
	Secondary	114	12.82
	University	3	0.34
Ethnicity	Adja and related	222	24.97
	Fon and related	450	50.62
	Dendi and related	188	21.15
	Gourmantché	15	1.69
	Peulh and related	3	0.34
	Yoa and related	2	0.22
	Yoruba and related	1	0.11
	Others	8	0.90

As for the 201 surveyed traditional practitioners, they included herbalists, phytotherapists and traditional healers (Table 4). Most of them were aged between 30 and 40 years and had nearly 15 years of experience. They were predominantly illiterate and belonged to the Dendi and related ethnic groups. The source of their knowledge was ancestral heritage.

**Table 4:** Socio-demographic characteristics of surveyed traditional healers

Variables	Categories	Total	Percentage (%)
Occupation	Herbalists	72	35.82
	Traditherapists	48	23.88
	Phytotherapists	80	39.80
	Others	1	0.50
Age	21–30 years old	30	14.93
	31–40 years old	73	36.32
	41–50 years old	60	29.85
	51–60 years old	38	18.91
Source of endogenous medicinal knowledge	Ancestral or family heritage	101	50.25
	Personal search	65	32.34
	Gift from God	25	12.44
	Training from a traditional medical practitioner	10	4.98
Years of experience	1–5 years old	10	4.98
	6–10 years old	44	21.89
	Age 11–15	57	28.36
	16–20 years	44	21.89
	21–25 years old	28	13.93
	26–30 years	11	5.47
	30 and over	7	3.48
Level of education	Illiterate	145	72.14
	Primary	44	21.89
	Secondary	11	5.47
	University	1	0.50
Ethnicity	Adja and related	27	13.43
	Fon and related	51	25.37
	Dendi and related	109	54.23
	Peulh and related	5	2.49
	Others	9	4.48

### Knowledge of respondents on malnutrition and associated pathologies

The knowledge of mothers and traditional practitioners on malnutrition (Table 5) showed that the majority of respondents (43.98%) had a good understanding of infantile malnutrition, including its causes, symptoms and consequences. They identified poverty, chronic diseases, infections, an unbalanced diet and insufficient food intake as the main causes. Signs such as excessive weight loss, bloated stomach, brittle hair and delayed growth were mentioned. The reported consequences included delayed growth, weakness in the child's body or immune system, the child takes longer to learn to move and speak, the child experiences difficulties in understanding and learning things, the child catches diseases like diarrhea, respiratory tract infections which can have symptoms such as cough, sore throat, fever, runny nose and difficulty breathing. Sometimes the child can die. However, their knowledge of associated pathologies was moderate.

**Table 5:** Knowledge of respondents on malnutrition

Variables	Categories	Total_Mothers looking after children	Percentage (%)_Mothers Babysitters	Total traditherapists	Percentage (%) traditherapists
Knowledge of the definition of malnutrition	Good	478	43.98	117	58.21
	Average	448	43.31	78	38.81
	Poor	119	12.71	6	2.99
Knowledge of the causes of malnutrition	Good	518	48.14	135	6.16
	Average	353	34.08	50	2.88
	Low	174	17.77	16	7.96
Knowledge of the consequences of malnutrition	Good	404	38.92	103	5.24
	Medium	395	36.45	71	3.32
	Low	246	24.63	27	1.43
Knowledge of the symptoms of malnutrition	Good	560	51.86	144	71.64
	Medium	363	34.98	52	25.87
	Low	122	13.16	5	2.49
Knowledge of pathologies associated with malnutrition	Good	285	25.53	58	28.86
	Medium	384	39.93	74	36.82
	Low	376	34.53	69	34.33

### Traditional management of infantile malnutrition in Benin

#### Informant Consensus Factor (ICF)

The Informant Consensus Factor (ICF) reflects the level of knowledge and collective understanding of medicinal plant use. In this study, the ICF for plants used in traditional malnutrition management is relatively high (ICF = 0.83), indicating consensus among respondents. Notably, mothers of children exhibit a higher ICF (0.91) compared to traditional healers (0.76) (Table 6).

**Table 6:** Result of the Informant Consensus Factor

	*N*	*N*_uc_	*N*_s_	ICF
Mothers of children	844	840	74	0.91
Traditional healers	201	211	52	0.76
All informants	1045	1051	126	0.83

### Medicinal recipes and plants involved in traditional treatment of malnutrition and associated pathologies

#### Medicinal recipes used by mothers of children

Surveyed mothers utilized 122 medicinal recipes, categorized as 71 single-plant recipes, 33 two-plant recipes, 13 three-plant recipes, 3 four-plant recipes and 2 five-plant recipes (Additional file 1: Table S7). These recipes involved a total of 74 medicinal plants (Additional file 2: Table S8). *Moringa oleifera, Phyllanthus amarus, Senna siamea, Carica papaya* and *Ocimum gratissimum* were highly cited in single-plant recipes. Two-plant combinations, such as *Senna siamea and Citrus aurantifolia*, and *Moringa oleifera and Glycine max*, were frequently mentioned. Notable three-plant associations included *Senna occidentalis, Moringa oleifera and Elaeis guineensis*, and *Carica papaya, Senna occidentalis and Pavetta crassipes*. *Moringa oleifera* was the most frequently cited and used in various recipes, followed by *Senna siamea, Carica papaya, Phyllanthus amarus* and *Ocimum gratissimum* (Additional file 3: Table S9).

#### Traditional practitioners’ medicinal recipes

Traditional practitioners recorded 52 medicinal plants in 71 recipes, including 23 single-plant recipes, 25 two-plant recipes, 19 three-plant recipes, 3 four-plant recipes and 1 five-plant recipe (Additional file 2: Table S8). Most cited recipes involved *Moringa oleifera* Lam. (single-plant) and its combinations with *Ficus platyphylla*, *Senna occidentalis, Elaeis guineensis* and *Carica papaya* (two- and three-plant recipes). Plants with the highest citation frequencies, significant contributions to recipes and a high-fidelity index were *Moringa oleifera Lam*., *Elaeis guineensis* Jacq., *Senna occidentalis* (L.) Link, *Sterculia setigera* Delile and *Carica papaya* L. (Additional file 3: Table S9).

#### Botanical families of medicinal plants

**Fig. 2:**
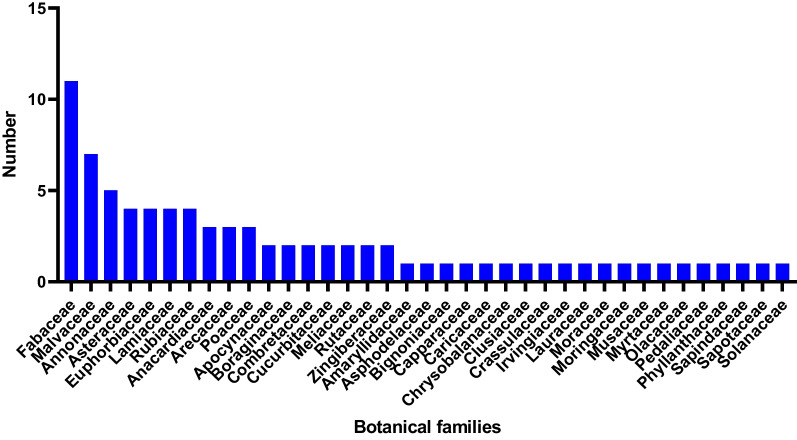
Botanical families of medicinal plants

Figure 2 provides information on the botanical families of the recorded plants. The most represented families are Fabaceae, Malvaceae and Annonaceae.

#### Parts of medicinal plants used in medicinal recipes

**Fig. 3:**
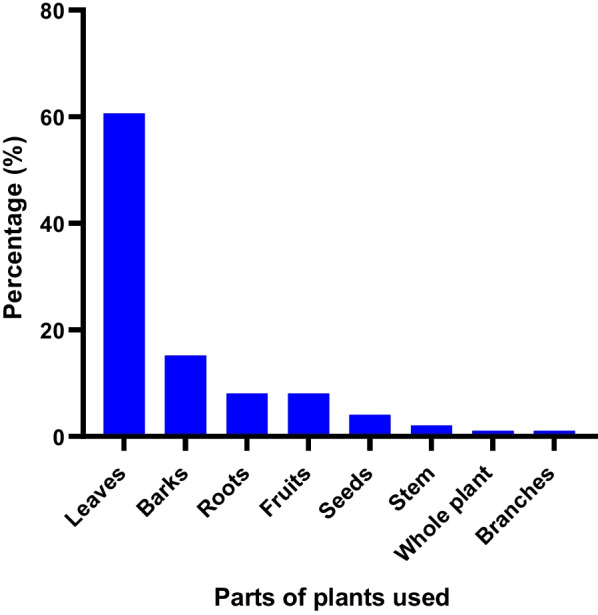
Parts of plants used in preparation of recipes

An analysis of the plant parts used in the remedies recorded shows that leaves were the most frequently used, from 48 species (60.60%). They were followed by bark (13 species, 15.15%), roots (7 species, 8.08%) and fruit (7 species, 8.08%) (Fig. 3). Other plant parts such as seeds, stems, the whole plant and branches were also used to prepare remedies, but only to a small extent. With regard to the state of the plant parts used in the preparation of remedies, the majority (90.1%) of remedies were made from freshly harvested plant parts, while a few preparations used dried plant parts (9.9%).

## Discussion

The aim of this study was to document knowledge and practices relating to the management of child malnutrition and associated pathologies, by targeting 844 Beninese mothers of children and 201 traditional practitioners. The approach to recruiting participants was transparent and inclusive. Close collaboration with local communities was established, working in partnership with local authorities, community leaders and health centers. This approach facilitated the integration of the study into the local context, fostering the trust and cooperation of the participating mothers and traditional healers. These two target groups come from different geographical regions and socioeconomic backgrounds in Benin. The use of traditional healers by mothers in cases of child malnutrition is a well-established practice. For example, a study in Ethiopia focusing on the qualitative analysis of care-seeking behavior of mothers of malnourished children confirmed this [36]. The findings of these authors highlighted the importance of understanding traditional healing practices within communities affected by child malnutrition. Therefore, by including traditional healers in research efforts, it is possible to achieve a more comprehensive understanding of local healthcare practices. In Ghana, there is also the possibility of using traditional healers to treat various spiritual and physical aspects of child malnutrition [37]. In India, this traditional approach to the management of child malnutrition is also practiced [38]. The dimension reported by Chaturvedi et al. [38] focused on infant massage with oil, which appears to be a potentially beneficial practice for children's nutrition.

These observations show that traditional ancestral knowledge can be used to enrich the child healthcare platform, demonstrating the importance of two medicines (modern and traditional) working together to tackle complex health challenges.

Our study revealed that the majority of participating women were married, illiterate, of Fon and Fon-related ethnic origin, and aged between 21 and 40. These data are comparable to those reported in Za-Kpota in central Benin and Zè in southern Benin [39]. The high illiteracy rate reflects the sociocultural realities of most Beninese populations [40]. This finding of widespread illiteracy raises important questions about access to education and its potential impact on decision-making, family health and other aspects of daily life.

Regarding the plurality of ethnicities, with a predominance of the Fon and related ethnic group, this underscores the importance of cultural and ethnic diversity in the study context. These data are in line with the findings of the report on the demographic survey carried out by INSAE (National Institute of Statistics and Economic Analysis) in 2013 [40]. This report indicates that in southern Benin, the dominant ethnic groups are the Fon and related groups (39.2%), the Adja and related groups (15.2%) and the Yoruba and related groups (14.5%). Finally, the specific age range of 21 to 40 years emphasizes that the survey focused on a specific period in women’s lives when motherhood and parenting are particularly relevant [39]. The second target of the study, namely traditional healers, was mostly aged 30 to 40, illiterate and of Dendi ethnicity and related groups, with ancestral heritage as the source of their knowledge. These same characteristics of respondents have been noted in other ethnobotanical studies carried out for various pathologies in Benin [41–45].

This highlights the importance of intergenerational transmission of traditional knowledge, emphasizing the cultural richness and ancestral heritage of the surveyed community [28]. These traditional healers had significant experience, around 15 years, indicating a profound understanding of traditional medicinal practices. This predisposes them to provide relevant information regarding the traditional management of infant malnutrition and associated pathologies [26].

Furthermore, respondents provided relevant data regarding their knowledge of infant malnutrition. Indeed, the majority (43.98%) of respondents had a good understanding of the meaning of infant malnutrition. This suggests that a significant portion of the studied population grasps fundamental aspects of malnutrition, such as stunting, low weight for height and low weight for age. These data are similar to those noted among mothers of children in the communes of Za-Kpota and Zè during the baseline survey of the integrated community nutrition and poverty alleviation project in Benin [39]. This solid knowledge base can serve as a favorable starting point for implementing initiatives to strengthen awareness of malnutrition and prevent associated pathologies.

The good understanding of the causes, symptoms and consequences of malnutrition by respondents is also a positive element. The identified causes, such as poverty, chronic illnesses, infections, unbalanced diet and food insufficiency, reflect an awareness of the multidimensional factors contributing to malnutrition [2, 46]. This underscores the importance of implementing malnutrition prevention programs that address these diverse aspects. The symptoms mentioned by respondents, such as excessive weight loss, bloating, brittle reddish hair and delayed growth, indicate recognition of the physical signs of malnutrition. This information is also supported by the mothers of children interviewed in eastern Ethiopia about child malnutrition, who reported similar causes and symptoms [36]. These data highlight the need for early detection and treatment of child malnutrition. However, the mention of moderate knowledge of conditions frequently associated with malnutrition highlights an area where further efforts may be needed to improve awareness.

Environmental problems linked to food and public health in the study area include poverty, lack of food, unhealthy diets, infectious diseases and lack of awareness [14, 15, 37]. Poverty is clearly identified as one of the causes of malnutrition, highlighting a major socioeconomic problem that hinders people's access to adequate food. Food insufficiency is also singled out, pointing to shortcomings in the availability and accessibility of food in the region studied. The finding of an unbalanced diet highlights concerns about the nutritional quality of the food available in the area. In addition, infections are mentioned as another cause of malnutrition, suggesting public health issues such as restricted access to healthcare and environmental conditions conducive to the spread of disease. Finally, the limited awareness of the conditions frequently associated with malnutrition highlights a deficit in the communication of nutrition and public health information within the community, which could pose additional challenges to the fight against this scourge. These aspects underline the need for integrated interventions aimed at improving access to adequate food, strengthening health systems and raising public awareness of nutrition and health issues.

Plant-based products are an invaluable aid in the management of malnutrition, due to their various contributions, mainly phytonutrients. In recent years, the demand for phytonutrients to supplement nutrition [47]. In this study, the mothers and traditional healers provided the homogeneous information’s about medicinal recipes used in traditional treatment of malnutrition and associated pathologies. Many medicinal recipes are used by both mothers of children and traditional healers in the context of managing infant malnutrition and associated pathologies. The study highlights the diversity of the medicinal recipes used, with a total of 122 recipes used by mothers and 71 by traditional healers for a total of 81 plants. This diversity of plants is greater than the 27 species of wild plants used as fruit or seasonal wild vegetables (in the case of other species) in the management of malnutrition [48]. This highlights that practices or approaches using medicinal plants to address malnutrition are shared by actors from different countries to meet these health challenges.

The recipes identified in this study aim to address various aspects such as appetite stimulation, essential nutrient intake, immune system strengthening, digestion improvement and the treatment of fever, diarrhea, convulsions and cough. This emphasizes the multifaceted approach through medicinal recipes proposed by the respondents, who also have a deep understanding of malnutrition and related pathologies. Moreover, recipes involving multiple medicinal plants aim to consider not only malnutrition but also any pathology associated with it. However, a meticulous scientific examination is necessary to attest to the effectiveness and safety of these plant combinations.

The study also highlights the most cited plants in the recipes, such as *Moringa oleifera., Adansonia digitate* L.*, Gymnanthemum amygdalinum* (Delile) Sch.Bip.*, Phyllanthus amarus, Senna siamae, Carica papaya, Ocimum gratissimum, Arachis hypogaea* L.*, Glycine max* (L.) Merr. Several studies have reported the nutritional potential of these plants [15, 17, 18]. Certain species, such as *Solanum macrocarpon* L. and others, share certain botanical families with the plants in this study [48]. Ojha et al. [49] identified several medicinal plants, including *Glycine max*, as food supplements with good sources of fat. In addition, these authors stated that in cases of stunted growth or to enhance the value of breast milk, the consumption of *Glycine max* and other plants is strongly recommended. Laleye et al*.* [18] evaluated the impact of porridge made from *Moringa oleifera* by administering it 5 times a week over 6 months. The results showed that 70% of the children and over 75% of the mothers found the porridge appetizing. A significant average weight gain of 1,720 g (*p *< 0.005) was observed in the intervention group at the end of the experiment, with a reduction in the prevalence of underweight of 1.33% (*p *> 0.05), and in the number of children suffering from acute malnutrition of 10.42% (*p *< 0.005). *Mentha* × *piperita* L.*,* identified in this study, is rich in menthyl acetate, menthone and menthol as the main phytoconstituents and methofuran, neomenthol, isomenthone, isorhoifolin, leutolin-7-O-glucoside and 1,8-cineole as other active constituents of mentha leaves. Thanks to its compounds, this plant has antioxidant, antimicrobial, insecticidal and anti-inflammatory activities, which could explain its involvement in the management of malnutrition and associated pathologies [50].

With its scientific name, *Arachis hypogaea*, identified in this study, peanuts contain numerous functional compounds such as proteins, fibers, polyphenols, antioxidants, vitamins and minerals which are very useful for improving the nutritional status of those suffering from malnutrition [51]. Its nutritional potential has been known for a very long time. The individual nutrients present in peanuts act through various mechanisms and can have synergistic effects to improve health. A study of over 15,000 people who consumed peanuts and peanut products showed higher levels of vitamin A, vitamin E, folate, magnesium, zinc, iron, calcium and dietary fiber than those who did not [52].

Red palm oil, extracted from *Elaeis guineensis* Jacq. as identified in this study, serves as the primary vegetable source of provitamin A carotenoids and is highly bioavailable due to its lipid structure and lack of a plant matrix [53]. In addition to its contribution to vitamin A, red palm oil provides lipids, often deficient and crucial for the biological efficacy of dietary provitamin A carotenoids. Moreover, red palm oil serves as a source of various antioxidants, including vitamin E and carotenoids other than vitamin A, which play a role in preventing cancer and other chronic diseases. Its nutrient-rich composition renders it a beneficial component for managing malnutrition [53].

*Gymnanthemum amygdalinum* is also one of the plants identified in this study. As a rich source of minerals and beneficial elements, this plant is considered a potential source of useful foods and medicines. Garba et Oviosa [54] revealed that *Gymnanthemum amygdalinum* can contribute to recommended dietary intakes of Fe (20%), Cu (27%), Mg (12%) and Ca (9%). It is also a good anti-anemic and anti-diabetic agent due to its high iron content. This makes it a good plant resource for managing malnutrition.

*Glycine max* (soya beans), another plant resource, has been known for its protein energy potential to improve nutritional status for ages. Several scientific studies have documented its nutritional properties. In Nigeria, for example, the use of soya beans both as soya milk and as “soyogi,” among other home formulations, provides high nutritional value and has long been strongly recommended for the prevention and management of malnutrition [55]. Agyenim-Boateng et al. [56] reported in a recent study the rich nutritional composition of soya beans (13.49% protein, 7.81% fatty acids, 2.47% soluble sugars, abundant mineral and micronutrient content), including folate (462. 27 μg FW) and carotenoids (3935.41 μg FW), isoflavone content ranged from 129.26 to 2359.35 μg/g FW, 115.57% folic acid and 11.60% zinc and concluded that soya beans meets the nutritional needs of most countries.

*Manihot esculenta* Crantz* (*Cassava) is a plant species rich in bioactive nutrients such as minerals, essential fatty acids and antioxidants. It contains eight minerals. It contains eight minerals, of which the K content was the highest, followed by Mg, Ca, P, Zn, Fe, Cu and Na [57]. This nutritional composition makes it ideal for use in the management of malnutrition.

*Carica papaya* L. is a fruit well known throughout the world. It is a rich source of vitamins, minerals and phytochemicals and a good source of nearby nutrients. Ugo et al. [58] revealed that *Carica papaya* contains fat (2.01%), ash (2.18%), protein (6.50%), crude fiber (3.10%), carbohydrates (29.20%), vitamin C (68.59 mg/100 g), beta-carotene (303, 55 mg/100 g), B1 (199.31 mg/100 g), B2 (295.63 mg/100 g) and vitamin E (39.78 mg/100 g), minerals—phosphorus (1971.17 mg/100 g), sodium (30.42 mg/100 g), potassium (80.13 mg/100 g), calcium (1086.53 mg/100 g) and chromium (31.10 mg/100 g). It also contains phytochemicals such as flavonoids (899.53 mg/100 g), alkaloids (1569.13 mg/100 g), saponins (898.07 mg/100 g) and tannins (310.50 mg/100 g). This nutritional potential helps to improve the nutritional status of malnourished children.

In addition, some of these plants have been used in the formulation of flour to correct nutritional deficiencies in malnourished children [15]. These data show that these plants are rich in trace elements that are useful for the proper nutrition of children under the age of five. On their own, the above plants have biological properties against most of the pathologies associated with malnutrition (anti-diarrheal, antitussive, antimicrobial properties, etc.). However, there is very little data on the effectiveness of plant-based recipes against both malnutrition and associated diseases. This study provides a database that can be used to explore the efficacy of medicinal recipes for treating child malnutrition and associated diseases.

## Conclusion

The study provides a significant database to explore the medicinal and nutritional properties of herbal medicinal recipes. The results highlight specific plants, such as *Moringa oleifera, Elaeis guineensis, Phyllanthus amarus and Carica papaya*, which stand out due to their high citation frequencies, contribution to recipes and high-fidelity index. These findings suggest a strong potential for the effective use of traditional herbal medicine in managing infant malnutrition and associated pathologies. Given that combinations of two or three plants are widely used in recorded medicinal recipes, it would be very useful to carry out scientific investigations into the synergistic effects of these plant combinations in order to determine their relevance to the treatment of child malnutrition and associated pathologies.

### Supplementary Information

**Additional file 1: Table S7.** Medicinal recipes used by mothers for the traditional management of infant malnutrition.

**Additional file 2: Table S8.** Medicinal recipes used by traditional therapists for the traditional treatment of child malnutrition.

**Additional file 3: Table S9.** Data on medicinal plants used by informants.

## Data Availability

Raw and treated data generated during study are available on the link https://docs.google.com/spreadsheets/d/14i61sy5FZkB5gdlsOjpgGW6b_dUdg4CP/edit?usp=sharing&ouid=104812263323222099631&rtpof=true&sd=true for the data about mothers of children; https://docs.google.com/spreadsheets/d/1mPQfDKwyMZWrZ1XgaYb-0kNWLM_dDDFl/edit?usp=sharing&ouid=104812263323222099631&rtpof=true&sd=true. for the data about traditional healers.

## Abbreviations

Cpr: Contribution of plant species to recipe
FAO: Food and Agriculture Organization
Fc: Frequency of medicinal plant citations
FI: Fidelity index
HIV/AIDS: Human immunodeficiency virus/acquired immunodeficiency syndrome
ICF: Informant Consensus Factor
INSAE: National Institute of Statistics and Economic Analysis
IQ: Intelligence Quotient
Ip: The number of informants who have used the given species to treat a specific category of diseases
Iu: The total number of surveyed informants
MICS: Multiple Indicator Cluster Surveys
*N*r: Number of recipes involving the plant species
*N*s: Number of plant species cited by informants in this category
*N*t: Total number of recipes
Nuc: Number of use citations in each informant category
RGPH: General Population and Housing Census
WFO: World Flora Online
WHO: World Health Organization
