# Supplementary material for: Knowledge and practices of traditional management of child malnutrition and associated pathologies in Benin
Source: J Ethnobiol Ethnomed. 2024 May 2;20:47. doi: 10.1186/s13002-024-00684-x (PMC11064319; doi:10.1186/s13002-024-00684-x)
Supplement: Supplementary file 1 — Additional file 1: Table S7. Medicinal recipes used by mothers for the traditional management of infant malnutrition. [file 13002_2024_684_MOESM1_ESM.docx]

**Additional information**

**Table 7.** Medicinal recipes used by mothers for the traditional management of infant malnutrition

| N° | Recipe type | Scientific names of plants | Plant organs | Plant use status | What treatment does the recipe target? | Preparation method | Criteria for use | Posology | Citation frequency |
| --- | --- | --- | --- | --- | --- | --- | --- | --- | --- |
| 1 | Monoplant recipe | *Moringa oleifera* Lam. | Leaves Barks Stems | It doesn't matter | Stimulation of appetite Fever Supply of essential nutrients Diarrhoea Convulsion Strengthening of the immune system Improvement of digestion Cough Diarrhoea | Infusion Decoction Trituration | Age Gender Child's status | Tadokpemi glass Bamboo glass | 31.96 |
| 2 | Monoplant recipe | *Phyllanthus amarus* Schumach. & Thonn | Leaves | Fresh | Supply of essential nutrients Stimulation of appetite Improved digestion Strengthened immune system Cough Diarrhea | Infusion | Child's condition | Tadokpemi glass | 7.36 |
| 3 | Monoplant recipe | *Senna siamea* (Lam.) H.S.Irwin & Barneby | Leaves Barks Fruits | Fresh | Stimulation of appetite Supply of essential nutrients Diarrhea Strengthening of the immune system Fever Diarrhea Cough Convulsion | Infusion Decoction Powder | Child's condition On the recommendation of the traditherapist or herbalist | Bamboo glass | 6.92 |
| 4 | Monoplant recipe | *Carica papaya* L. | Leaves | Fresh | Fever | Infusion/Trituration/Maceration | On the recommendation of the traditherapist or herbalist | Bamboo glass | 6.19 |
| 5 | Monoplant recipe | *Ocimum gratissimum* L. | Leaves | Whatever | Supply of essential nutrients Stimulation of appetite Strengthening of the immune system Diarrhea Fever Improved digestion Cough Convulsion | Infusion Trituration | Child's condition Age | Coffee spoon Tadokpemi glass Bamboo glass | 5.60 |
| 6 | Monoplant recipe | *Adansonia digitata* L. | Bark | Dry Fresh Whatever | Stimulation of appetite Supply of essential nutrients Strengthening of the immune system, Improvement of digestion, Fever, | Infusion | Age, On the recommendation of the traditherapist or herbalist | Bamboo glass | 4.42 |
| 7 | Monoplant recipe | *Khaya senegalensis* (Desr.) A.Juss. | Bark | Doesn't matter | Stimulation of appetite Supply of essential nutrients Strengthening of the immune system Diarrhoea | Infusion Trituration Decoction | Child's condition On the recommendation of the therapist or herbalist Age | Tadokpemi glass Bamboo glass | 3.83 |
| 8 | Monoplant recipe | *Gymnanthemum amygdalinum* (Delile) Sch.Bip. | Leaves, roots | Fresh | Intake of essential nutrients Appetite stimulation Digestion improvement Diarrhea Fever Immune system strengthening Cough Convulsion | Decoction | Child's condition | Soup spoon Tadokpemi glass Soup spoon | 3.83 |
| 9 | Monoplant recipe | *Vitellaria paradoxa* C.F.Gaertn. | Leaves | Dry | Stimulation of appetite Supply of essential nutrients Fever Improved digestion Diarrhea | Infusion | Child's condition On the recommendation of the herbalist or traditherapists | Bamboo glass | 2.21 |
| 10 | Monoplant recipe | *Anonychium africanum* (Guill. & Perr.) C.E.Hughes & G.P.Lewis | Leaves | Fresh | Supply of essential nutrients Fever Diarrhea Appetite stimulation Digestion improvement Cough Convulsion | Infusion | Age sex On traditherapists or herbalist's recommendation | Tadokpemi glass | 2.06 |
| 11 | Monoplant recipe | *Momordica charantia* L*.* | Leaves Barks Roots | Fresh Any | Essential nutrients Appetite stimulation Fever Cough Diarrhea Immune system boost Digestion enhancement | Infusion Maceration | Child's condition | Coffee spoon Tadokpemi glass | 1.77 |
| 12 | Monoplant recipe | *Mangifera indica* L. | Leaves | Doesn't matter | Immune system boost Fever Diarrhea Essential Nutrients Diarrhea Convulsion Appetite stimulation Convulsion Fever Cough Digestion enhancement | Trituration | Sex Age | Coffee spoon Soup spoon | 1.62 |
| 13 | Monoplant recipe | *Citrus × aurantiifolia* (Christm.) Swingle | Leaves | Doesn't matter | Supply of essential nutrients Stimulation of appetite Improved digestion Diarrhoea Fever Convulsion Cough Diarrhoea Immune system boost Fever Convulsion | Infusion Powder | Child's condition | Soup spoon | 1.33 |
| 14 | Monoplant recipe | *Paullinia pinnata* L. | Leaves Roots | Fresh | Supply of essential nutrients Stimulation of appetite Improvement of digestion Fever Strengthening of the immune system Diarrhea Cough | Infusion | Age Sex | Tadokpemi glass Bamboo glass | 1.33 |
| 15 | Monoplant recipe | *Senna occidentalis* (L.) Link | Leaves | Doesn't matter | Supply of essential nutrients Fever Cough Convulsion Immune system strengthening Appetite stimulation Digestion improvement | Infusion | On the recommendation of the therapist or herbalist Child's condition | Tadokpemi glass Bamboo glass | 1.18 |
| 16 | Monoplant recipe | *Allium cepa* L. | Leaves | Doesn't matter | Supply of essential nutrients Stimulation of appetite Improved digestion Strengthening of the immune system Fever Cough | Infusion Decoction Powder | Child's condition Sex | Soup spoon | 1.03 |
| 17 | Monoplant recipe | *Arachis hypogaea* L. | Leaves Barks Roots | Doesn't matter | Supply of essential nutrients Fever Diarrhea Stimulation of appetite Improved digestion Strengthened immune system Fever Cough | Infusion/Trituration/Maceration | Child's condition | Tadokpemi glass Bamboo glass Soup spoon | 0.88 |
| 18 | Monoplant recipe | *Croton gratissimus* Burch. | Leaves | Doesn't matter | Supply of essential nutrients Stimulation of appetite Convulsion Fever Cough Improved Digestion Convulsion | Infusion | On the recommendation of the therapist or herbalist | Tadokpemi glass Bamboo glass | 0.74 |
| 19 | Monoplant recipe | *Erythrina senegalensis* DC. | Leaves | Fresh | Supply of essential nutrients Stimulation of appetite Fever Convulsion Improvement of digestion Strengthening of the immune system | Infusion | Child's condition | Coffee spoon Tadokpemi glass Bamboo glass | 0.74 |
| 20 | Monoplant recipe | *Pavetta corymbosa* (DC.) F.N.Williams | Leaves | Fresh | Supply of essential nutrients Immune system boost Digestion improvement Fever Appetite stimulation | Infusion | Age Child's condition | Coffee spoon | 0.74 |
| 21 | Monoplant recipe | *Psidium guajava* L. | Leaves Barks | Fresh | Diarrhoea Appetite stimulation Essential nutrient intake Fever Cough | Infusion, Decoction | Child's condition On traditherapist's or herbalist's recommendation | Bamboo glass | 0.74 |
| 22 | Monoplant recipe | *Pterocarpus erinaceus* Poir. | Seeds | Doesn't matter | Supply of essential nutrients Stimulation of appetite Strengthening of the immune system Fever Improved digestion Cough Diarrhea | Powder Infusion Maceration Trituration | Child's condition On the recommendation of the traditherapist or herbalist | Coffee spoon Bamboo glass Tadokpemi glass | 0.74 |
| 23 | Monoplant recipe | *Calotropis procera* (Aiton) Dryand. | Leaves Barks Roots | Fresh Whatever | Supply of essential nutrients Fever Convulsion Diarrhea | Infusion | Child's condition | Coffee spoon Bamboo glass Tadokpemi glass | 0.59 |
| 24 | Monoplant recipe | *Pavetta crassipes* K.Schum. | Bark | Whatever | Fever Appetite stimulation | Infusion | On traditherapist's or herbalist's recommendation | Bamboo glass | 0.59 |
| 26 | Monoplant recipe | *Musa × paradisiaca* L. | Leaves | Dry | Supply of essential nutrients Appetite stimulation Fever Improved digestion | Infusion | Child's condition On traditherapist's or herbalist's recommendation | Bamboo glass | 0.59 |
| 27 | Monoplant recipe | *Solanum macrocarpon* L. | Leaves | Fresh | Stimulation of appetite Supply of essential nutrients Fever Strengthening of the immune system Improvement of digestion | Infusion | Child's condition On traditherapist's or herbalist's recommendation | Others | 0.59 |
| 29 | Monoplant recipe | *Cymbopogon citratus* (DC.) Stapf | Leaves | Not important | Supply of essential nutrients Stimulation of appetite Fever Improvement of digestion | Infusion, Decoction, Drink | Age Sex | Glass tadokpemi Others | 0.44 |
| 30 | Monoplant recipe | *Mesosphaerum suaveolens* (L.) Kuntze | Leaves | Doesn't matter | Supply of essential nutrients Stimulation of appetite Strengthening of the immune system Convulsion Diarrhea Fever | Infusion | Child's condition | Coffee spoon Tadokpemi glass Bamboo glass | 0.44 |
| 31 | Monoplant recipe | *Spondias mombin* L. | Leaves | Doesn't matter | Essential nutrients Appetite stimulation Fever Cough Convulsion | Infusion | Child's condition | Glass tadokpemi | 0.44 |
| 32 | Monoplant recipe | *Aloe buettneri* A.Berger | Leaves | Fresh | Fever | Maceration | Child's condition | Coffee spoon Bamboo glass | 0.29 |
| 33 | Monoplant recipe | *Annona senegalensis* Pers. | Leaves Fruit | Fresh | Stimulation of appetite Improved digestion Supply of essential nutrients | Infusion | Child's condition | Bamboo glass | 0.29 |
| 34 | Monoplant recipe | *Crateva adansonii* DC. | Leaves Barks | Fresh Any | Supply of essential nutrients Immune system boost Convulsion Fever | Infusion | Child's condition | Coffee spoon Bamboo glass Tadokpemi glass | 0.29 |
| 35 | Monoplant recipe | *Glycine max* (L.) Merr. | Seeds | Dry | Supply of essential nutrients Immune system support | Powder | Child's age | Coffee spoon | 0.29 |
| 36 | Monoplant recipe | *Hibiscus sabdariffa* L. | Leaves | Not important | Supply of essential nutrients Convulsion Improved digestion | Infusion | Child's status | Tadokpemi glass Soup spoon Coffee spoon | 0.29 |
| 37 | Monoplant recipe | *Landolphia dulcis* (Sabine ex G.Don) Pichon | Fruit | Doesn't matter | Immune system boost Digestion enhancement Appetite stimulation Essential nutrients Fever Diarrhea Cough | Infusion Trituration | Child's age Sex | Soup spoon | 0.29 |
| 38 | Monoplant recipe | *Launaea taraxacifolia* (Willd.) Amin ex C.Jeffrey | Leaves | Fresh | Supply of essential nutrients Stimulation of appetite Fever Diarrhea Improved digestion | Trituration | Child's status | Tadokpemi glass | 0.29 |
| 39 | Monoplant recipe | *Ocimum americanum* L. | Leaves, roots | Doesn't matter | Strengthening of the immune system Cough Supply of essential nutrients Fever | Infusion Trituration | Sex Child's condition | Bamboo glass | 0.29 |
| 40 | Monoplant recipe | *Oxytenanthera abyssinica* (A.Rich.) Munro | Leaves | Fresh | Stimulation of appetite Fever Diarrhoea Supply of essential nutrients Strengthening of the immune system | Infusion | Child's condition On the recommendation of the traditherapist or herbalist | Bamboo glass | 0.29 |
| 41 | Monoplant recipe | *Sterculia setigera* Delile | Bark | Fresh | Improve digestion Provide essential nutrients Stimulate appetite | Infusion Maceration Others | Child's condition Age Sex | Coffee spoon Soup spoon | 0.29 |
| 43 | Monoplant recipe | *Vachellia seyal* (Delile) P.J.H.Hurter | Leaves | Doesn't matter | Supply of essential nutrients Stimulation of appetite Fever Convulsion | Infusion | Child's condition On recommendation of traditherapist or herbalist | Tadokpemi glass | 0.29 |
| 44 | Monoplant recipe | *Adansonia digitata* L. | Leaves Fruits | Dry | Diarrhea | Trituration | Child's condition Age Sex | Coffee spoon | 0.15 |
| 45 | Monoplant recipe | *Adansonia digitata* L. | Seeds | Dry | Fever | Trituration | Child's condition | Coffee spoon Tadokpemi glass Bamboo glass | 0.15 |
| 46 | Monoplant recipe | *Ageratum conyzoides* L. | Leaves | Fresh | Stimulation of appetite Improvement of digestion | Trituration | Child's condition | Tadokpemi glass | 0.15 |
| 47 | Monoplant recipe | *Azadirachta indica* A.Juss. | Leaves | Fresh | Fever Other | Infusion | Child's condition | Bamboo glass | 0.15 |
| 48 | Monoplant recipe | *Rhodognaphalon brevicuspe* (Sprague) Roberty | Leaves | Fresh | Supply of essential nutrients Stimulation of appetite Improvement of digestion Fever Cough Diarrhea | Infusion Maceration | Child's condition | Coffee spoon | 0.15 |
| 49 | Monoplant recipe | *Sesamum radiatum* Thonn. ex Hornem. | Leaves | Fresh | Appetite stimulation | Infusion | On the recommendation of the herbalist State of the child | Bamboo glass | 0.15 |
| 50 | Monoplant recipe | *Citrus maxima* (Burm.) Merr. | Fruits Others | Fresh | Appetite stimulation | Infusion | On the recommendation of the traditherapist or herbalist Child's condition | Bamboo glass | 0.15 |
| 51 | Monoplant recipe | *Cleistopholis patens* (Benth.) Engl. & Diels | Leaves | No matter | Fever Diarrhea | Decoction | On the recommendation of the traditherapist or herbalist | Bamboo glass Soup spoon Coffee spoon | 0.15 |
| 52 | Monoplant recipe | *Cocos nucifera* L. | Leaves | Whatever | Stimulates appetite Improves digestion Strengthens the immune system Diarrhea | Powder Other | Child's condition | Coffee spoon | 0.15 |
| 53 | Monoplant recipe | *Combretum collinum* Fresen. | Seeds | Whatever | Appetite stimulation | Infusion | On the recommendation of the traditherapist or herbalist Child's condition | Bamboo glass | 0.15 |
| 54 | Monoplant recipe | *Ehretia cymosa* Thonn. | Leaves | Fresh | Supply of essential nutrients Strengthening of the immune system Cough Convulsion | Infusion | Child's condition | Coffee spoon Tadokpemi glass Bamboo glass | 0.15 |
| 55 | Monoplant recipe | *Elaeis guineensis* Jacq. | Twigs Fruits | Fresh | Supply of essential nutrients | Infusion | Age Sex | Coffee spoon Tadokpemi glass Bamboo glass | 0.15 |
| 56 | Monoplant recipe | *Euphorbia hirta* L. | Leaves | Fresh | Supply of essential nutrients Immune system boost Digestion improvement | Infusion | Child's condition Age | Coffee spoon | 0.15 |
| 57 | Monoplant recipe | *Ficus platyphylla* Delile | Leaves Barks Stems | Doesn't matter | Essential nutrients Appetite stimulation Digestion improvement Fever Immune system strengthening | Water Honey Oil | On tradithérapeute's or herbalist's recommendation | Tadokpemi glass Bamboo glass | 0.15 |
| 58 | Monoplant recipe | *Heliotropium indicum* L. | Leaves | Fresh Whatever | Supply of essential nutrients Stimulation of appetite Strengthening of the immune system | Maceration Trituration | Child's condition | Coffee spoon Soup spoon | 0.15 |
| 59 | Monoplant recipe | *Hibiscus cannabinus* L*.* | Leaves Barks | Fresh | Supply of essential nutrients Immune system boost Diarrhea | Infusion | Child's condition | Tadokpemi glass | 0.15 |
| 60 | Monoplant recipe | *Hyphaene thebaica* (L.) Mart*.* | Seeds | Any | Fever | Infusion | Child's condition Age Sex | Coffee spoon Soup spoon | 0.15 |
| 61 | Monoplant recipe | *Senna italica* Mill. | Leaves | Fresh | Supply of essential nutrients Improvement of digestion Stimulation of appetite Strengthening of the immune system | Infusion | Child's condition Age | Coffee spoon | 0.15 |
| 62 | Monoplant recipe | *Irvingia gabonensis* (Aubry-Lecomte ex O'Rorke) Baill. | Leaves Barks | Dry | Supply of essential nutrients Stimulation of appetite Improved digestion | Infusion | Age Sex Other | Tadokpemi glass | 0.15 |
| 65 | Monoplant recipe | *Macrosphyra longistyla* (DC.) Hook.f. ex Hiern | Bark | Fresh | Appetite stimulation Fever | Decoction | Child's condition | Tadokpemi glass | 0.15 |
| 66 | Monoplant recipe | *Momordica balsamina* L. | Leaves | Fresh | Improved digestion Strengthened immune system Cough Diarrhea Fever Supply of essential nutrients | Infusion Trituration | Child's condition | Coffee spoon | 0.15 |
| 67 | Monoplant recipe | *Newbouldia laevis* (P.Beauv.) Seem. ex Bureau | Leaves | Fresh | Supply of essential nutrients Fever Cough Diarrhea | Infusion | Child's condition | Tadokpemi glass | 0.15 |
| 69 | Monoplant recipe | *Piliostigma thonningii* (Schumach.) Milne-Redh. | Leaves | No matter | Diarrhea Supply of essential nutrients Immune system boost Fever | Infusion | Child's condition | Coffee spoon | 0.15 |
| 70 | Monoplant recipe | *Moringa oleifera* Lam. | Leaves | Fresh | Supply of essential nutrients Stimulation of appetite Improved digestion Strengthened immune system Fever Diarrhoea | Infusion | Child's condition | Bamboo glass | 0.74 |
| 71 | Monoplant recipe | *Cymbopogon citratus* (DC.) Stapf | Leaves | Doesn't matter | Supply of essential nutrients Stimulation of appetite Improved digestion Strengthened immune system Fever Diarrhea | Infusion | Child's condition | Bamboo glass | 0.15 |
| 72 | Recipe with 2 plants | *Senna siamea* (Lam.) H.S.Irwin & Barneby | Leaves | Dry | Stimulates appetite Improves digestion Fever | Infusion | Child's condition | Bamboo glass | 8.70 |
|  |  | *Citrus × aurantiifolia* (Christm.) Swingle | Fruit | Fresh Dry Any |  |  |  |  |  |
| 73 | Recipe with 2 plants | *Spondias mombin* L*.* | Leaves | Fresh | Stimulation of appetite Fever Improved digestion Fever Diarrhea | Maceration | Child's condition On traditherapist's or herbalist's recommendation | Bamboo glass | 6.52 |
|  |  | *Cocos nucifera* L. | Fruit | Any |  |  |  |  |  |
| 74 | Recipe with 2 plants | *Moringa oleifera* Lam. | Leaves | Fresh | Supply of essential nutrients Improved digestion Stimulation of appetite Strengthening of the immune system. | Decoction, Infusion | Child's condition Age | Coffee spoon | 4.35 |
|  |  | *Glycine max* (L.) Merr. | Grain | dry |  |  |  |  |  |
| 75 | Recipe with 2 plants | *Senna occidentalis* (L.) Link | Leaves | Any | Supply of essential nutrients Improved digestion Strengthened immune system Fever Convulsion Cough | Infusion Trituration | Child's condition | Soup spoon | 435 |
|  |  | *Khaya senegalensis* (Desr.) A.Juss. | Bark | Doesn't matter |  |  |  |  |  |
| 76 | Recipe with 2 plants | *Vitellaria paradoxa* C.F.Gaertn. | Leaves | Dry | Supply of essential nutrients Stimulation of appetite Fever Strengthening of the immune system Improvement of digestion | Infusion | Child's condition | Bamboo glass | 4.35 |
|  |  | *Musa × paradisiaca* L. | Leaves | Dry |  |  |  |  |  |
| 77 | Recipe with 2 herbs | *Sterculia setigera* Delile | Bark | Doesn't matter | Supply of essential nutrients Stimulation of appetite Improved digestion Strengthened immune system Fever | Infusion | On the recommendation of the therapist or herbalist | Bamboo glass | 4.35 |
|  |  | *Terminalia leiocarpa* (DC.) Baill. | Leaves Barks | Doesn't matter |  |  |  |  |  |
| 78 | Recipe with 2 herbs | *Sterculia setigera* Delile | Barks | Doesn't matter | Essential nutrients Appetite stimulation Digestion enhancement Immune system strengthening Fever | Infusion | On the recommendation of the traditherapist or herbalist | Bamboo glass | 4.35 |
|  |  | *Pavetta crassipes* K.Schum. | Leaves Barks | Doesn't matter |  |  |  |  |  |
| 79 | Recipe with 2 herbs | *Moringa oleifera* Lam*.* | Leaves Seeds | Doesn't matter | Supply of essential nutrients Improved digestion Strengthened immune system | Infusion Trituration | Child's condition Age Sex | Coffee spoon | 4.35 |
|  |  | *Senna occidentalis* (L.) Link | Leaves Roots Stems Flowers Seeds Branches | Fresh |  |  |  |  |  |
| 80 | Recipe with 2 herbs | *Moringa oleifera* Lam. | Leaves | Doesn't matter | Supply of essential nutrients Stimulation of appetite Improved digestion Strengthened immune system | Infusion | On traditherapist's or herbalist's recommendation | Bamboo glass | 4.35 |
|  |  | *Elaeis guineensis* Jacq. | Bark | Doesn't matter |  |  |  |  |  |
| 81 | Recipe with 2 herbs | *Ocimum gratissimum* L. | Leaves Roots | Fresh | Stimulation of appetite Supply of essential nutrients Improvement of digestion Strengthening of the immune system | Fever Supply of essential nutrients | On the recommendation of the therapist or herbalist | Age Sex | 4.35 |
|  |  | *Vitellaria paradoxa* C.F.Gaertn. | Leaves | Dry |  |  |  |  |  |
| 82 | Recipe with 2 herbs | *Anacardium occidentale* L. | Bark | Doesn't matter | Supply of essential nutrients Stimulation of appetite Improved digestion Strengthened immune system Fever Cough | Infusion Powder | Child's condition Age Sex | Soup spoon | 2.17 |
|  |  | *Landolphia dulcis* (Sabine ex G.Don) Pichon | Fruits | Doesn't matter |  |  |  |  |  |
| 83 | Recipe with 2 herbs | *Gymnanthemum amygdalinum* (Delile) Sch.Bip. | Seeds | It doesn't matter | Essential nutrients Fever Diarrhea | Decoction | Child's condition | Bamboo glass | 2.17 |
|  |  | *Senna occidentalis* (L.) Link | Leaves | Doesn't matter |  |  |  |  |  |
| 84 | Recipe with 2 herbs | *Moringa oleifera* Lam. | Leaves | Fresh | Essential nutrients Fever Diarrhea | Decoction | Child's condition | Bamboo glass | 2.17 |
|  |  | *Mangifera indica* L. | Leaves | Doesn't matter |  |  |  |  |  |
| 85 | Recipe with 2 herbs | *Ocimum gratissimum* L. | Leaves Barks | Fresh Whatever | Essential nutrients Diarrhea | Decoction Powder Maceration | Child's condition | Coffee spoon | 2.17 |
|  |  | *Citrus × aurantiifolia* (Christm.) Swingle | Fruit | Doesn't matter Fresh |  |  |  |  |  |
| 86 | Recipe with 2 herbs | *Pavetta crassipes* K.Schum. | Bark | Doesn't matter | Supply of essential nutrients Diarrhea | Infusion | On the recommendation of the traditherapist or herbalist | Bamboo glass | 2.17 |
|  |  | *Carica papaya* L. | Leaves | Doesn't matter |  |  |  |  |  |
| 88 | Recipe with 2 herbs | *Khaya senegalensis* (Desr.) A.Juss. | Leaves Bark | Whatever | Supply of essential nutrients Improved digestion Fever Diarrhea | Infusion | On the recommendation of the herbalist or therapist | Bamboo glass | 2.17 |
|  |  | *Senna occidentalis* (L.) Link | Leaves | Whatever |  |  |  |  |  |
| 89 | Recipe with 2 herbs | *Khaya senegalensis* (Desr.) A.Juss. | Leaves Barks | Whatever | Supply of essential nutrients Stimulates appetite Improves digestion Strengthens the immune system | Infusion | On the recommendation of the herbalist or herbalist | Bamboo glass | 2.17 |
|  |  | *Pavetta crassipes* K.Schum. | Bark | Whatever |  |  |  |  |  |
| 90 | Recipe with 2 herbs | *Carica papaya* L. | Leaves | Whatever | Supply of essential nutrients Stimulation of appetite Improved digestion Strengthened immune system | Infusion | On traditherapist's or herbalist's recommendation | Bamboo glass | 2.17 |
|  |  | *Moringa oleifera* Lam. | Leaves | Doesn't matter |  |  |  |  |  |
| 91 | Recipe with 2 herbs | *Anonychium africanum* (Guill. & Perr.) C.E.Hughes & G.P.Lewis | Leaves | Fresh | Essential nutrients Appetite stimulation | Infusion | Age Sex | Tadokpemi glass | 2.17 |
|  |  | *Vitellaria paradoxa* C.F.Gaertn. | Leaves | Fresh |  |  |  |  |  |
| 92 | Recipe with 2 herbs | *Piliostigma thonningii* (Schumach.) Milne-Redh. | Leaves Barks Roots | Fresh | Essential nutrients Fever Diarrhea | Infusion | Child's condition | Tadokpemi glass | 2.17 |
|  |  | *Crateva adansonii* DC. | Leaves Barks | Fresh |  |  |  |  |  |
| 93 | Recipe with 2 herbs | *Heliotropium indicum* L. | Leaves | Any | Supply of essential nutrients Stimulation of appetite Improved digestion Diarrhoea | Infusion | Child's condition | Tadokpemi glass | 2.17 |
|  |  | *Moringa oleifera* Lam. | Leaves | Doesn't matter |  |  |  |  |  |
| 94 | Recipe with 2 herbs | *Moringa oleifera* Lam. | Leaves | Fresh | Diarrhea | Infusion | Child's condition | Tadokpemi glass | 2.17 |
|  |  | *Cassytha filiformis* L. | Leaves | Fresh |  |  |  |  |  |
| 95 | Recipe with 2 herbs | *Momordica charantia* L. | Leaves | Fresh | Fever Convulsion Cough Other Diarrhea | Infusion | Child's condition | Coffee spoon | 2.17 |
|  |  | *Moringa oleifera* Lam. | Roots | Fresh Dry |  |  |  |  |  |
| 96 | Recipe with 2 herbs | *Vitellaria paradoxa* C.F.Gaertn. | Leaves | Dry | Stimulation of appetite Supply of essential nutrients Strengthening of the immune system Fever Convulsion Improvement of digestion | Infusion | Child's condition On the recommendation of the traditherapist or herbalist | Bamboo glass | 2.17 |
|  |  | *Paullinia pinnata* L. | Leaves | Dry |  |  |  |  |  |
| 97 | Recipe with 2 herbs | *Glycine max* L. | Seeds | Not important | Essential nutrients Digestion enhancement Immune system strengthening Diarrhea | Powder | Child's condition Age Sex | Coffee spoon | 2.17 |
|  |  | *Combretum collinum* Fresen. | Seeds | No matter |  |  |  |  |  |
| 98 | Recipe with 2 herbs | *Parinari curatellifolia* Planch. ex Benth. | Leaves Barks | Doesn't matter | Supply of essential nutrients Fever Diarrhea Appetite stimulation | Decoction | Child's condition | Bamboo glass | 2.17 |
|  |  | *Senna siamea* (Lam.) H.S.Irwin & Barneby | Leaves | Fresh |  |  |  |  |  |
| 99 | Recipe with 2 herbs | *Sterculia setigera* Delile | Barks | Doesn't matter | Supply of essential nutrients Stimulation of appetite Improved digestion Strengthened immune system | Infusion | On the recommendation of the therapist or herbalist | Bamboo glass | 2.17 |
|  |  | *Moringa oleifera* Lam. | Leaves | Doesn't matter |  |  |  |  |  |
| 100 | Recipe with 2 herbs | *Pterocarpus erinaceus* Poir. | Bark | Doesn't matter | Supply of essential nutrients Improved digestion Fever Cough Diarrhea Strengthened immune system | Infusion | Child's condition | Soup spoon Coffee spoon | 2.17 |
|  |  | *Carica papaya* L. | Leaves | Doesn't matter |  |  |  |  |  |
| 101 | Recipe with 2 herbs | *Allium cepa* L. | Leaves | Fresh | Supply of essential nutrients Improves digestion Stimulates appetite | Trituration | Child's condition Age Sex | Soup spoon | 2.17 |
|  |  | *Moringa oleifera* Lam. | Leaves | Doesn't matter |  |  |  |  |  |
| 102 | Recipe with 2 herbs | *Moringa oleifera* Lam*.* | Leaves | Doesn't matter | Supply of essential nutrients Improved digestion Stimulation of appetite | Infusion | On the recommendation of the therapist or herbalist | Bamboo glass | 2.17 |
|  |  | *Elaeis guineensis* Jacq. | Bark | Doesn't matter |  |  |  |  |  |
| 103 | Recipe with 2 herbs | *Senna siamea* (Lam.) H.S.Irwin & Barneby | Leaves | Fresh | Appetite stimulation Digestion improvement Fever | Infusion | Age Sex | Tadokpemi glass | 2.17 |
|  |  | *Spondias mombin* L. | Leaves | Fresh |  |  |  |  |  |
| 104 | Recipe with 2 herbs | *Anonychium africanum* (Guill. & Perr.) C.E.Hughes & G.P.Lewis | Leaves | Fresh | Stimulation of appetite Supply of essential nutrients Improvement of digestion Strengthening of the immune system | Infusion | Age Sex | Tadokpemi glass | 2.17 |
|  |  | *Vitellaria paradoxa* C.F.Gaertn. | Leaves | Dry |  |  |  |  |  |
| 105 | Recipe with 2 herbs | *Senna occidentalis* (L.) Link | Leaves | Doesn't matter | Supply of essential nutrients Stimulates appetite Improves digestion Fever Diarrhea | Infusion | On traditherapist's or herbalist's recommendation | Bamboo glass | 5.56 |
|  |  | *Elaeis guineensis* Jacq. | Bark | Whatever |  |  |  |  |  |
| 106 | Recipe with 3 herbs | *Senna occidentalis* (L.) Link | Leaves | Any | Stimulation of appetite Supply of essential nutrients Improvement of digestion Strengthening of the immune system | Infusion | On the recommendation of the therapist or herbalist | Bamboo glass | 27.78 |
|  |  | *Moringa oleifera* Lam. | Leaves | Doesn't matter |  |  |  |  |  |
|  |  | *Elaeis guineensis* Jacq. | Leaves Bark | Doesn't matter |  |  |  |  |  |
| 107 | Recipe with 3 herbs | *Moringa oleifera* Lam. | Leaves | Doesn't matter | Supply of essential nutrients Stimulation of appetite Improved digestion Strengthened immune system | Infusion | On the recommendation of the herbalist or herbalist | Bamboo glass | 11.11 |
|  |  | *Elaeis guineensis* Jacq. | Bark | Doesn't matter |  |  |  |  |  |
|  |  | *Sterculia setigera* Delile | Bark | Whatever |  |  |  |  |  |
| 108 | Recipe with 3 herbs | *Carica papaya* L. | Leaves | Whatever | Supply of essential nutrients Stimulation of appetite Improved digestion Strengthened immune system Diarrhea | Infusion | On recommendation of traditherapist or herbalist | Bamboo glass | 5.56 |
|  |  | *Senna occidentalis* (L.) Link | Leaves | Whatever |  |  |  |  |  |
|  |  | *Pavetta crassipes* K.Schum. | Bark | Doesn't matter |  |  |  |  |  |
| 109 | Recipe with 3 herbs | *Manihot esculenta* Crantz | Leaves | Fresh | Supply of essential nutrients Appetite stimulation | Infusion | Age Sex | Tadokpemi glass | 5.56 |
|  |  | *Carica papaya* L. | Leaves | Fresh |  |  |  |  |  |
|  |  | *Allium cepa* L. | Fruit | fresh |  |  |  |  |  |
| 110 | Recipe with 3 herbs | *Senna siamea* (Lam.) H.S.Irwin & Barneby | Leaves | Doesn't matter | Supply of essential nutrients Stimulation of appetite Improved digestion Fever | Infusion | Child's condition | Bamboo glass | 5.56 |
|  |  | *Elaeis guineensis* Jacq. | Bark | Doesn't matter |  |  |  |  |  |
|  |  | *Moringa oleifera* Lam. | Leaves | Doesn't matter |  |  |  |  |  |
| 111 | Recipe with 3 herbs | *Senna occidentalis* (L.) Link | Leaves | Whatever | Supply of essential nutrients Stimulation of appetite Improvement of digestion Strengthening of the immune system Fever | Infusion | On traditherapist's or herbalist's recommendation | Bamboo glass | 5.56 |
|  |  | *Sterculia setigera* Delile | Bark | Whatever |  |  |  |  |  |
|  |  | *Pavetta crassipes* K.Schum. | Leaves Bark | Doesn't matter |  |  |  |  |  |
| 112 | Recipe with 3 herbs | *Vitellaria paradoxa* C.F.Gaertn. | Leaves | Dry Whatever | Convulsion Immune system boost Diarrhea | Decoction | Child's condition | Tadokpemi glass Soup spoon | 5.56 |
|  |  | *Paullinia pinnata* L. | Leaves | Doesn't matter |  |  |  |  |  |
| 113 | Recipe with 3 herbs | *Elaeis guineensis* Jacq. | Bark | Doesn't matter | Essential nutrients Stimulate appetite Improve digestion Strengthen the immune system Fever | Infusion | On traditherapist's or herbalist's recommendation | Bamboo glass | 5.56 |
|  |  | *Sterculia setigera* Delile | Barks | Doesn't matter |  |  |  |  |  |
|  |  | *Pavetta crassipes* K.Schum. | Leaves Bark | Doesn't matter |  |  |  |  |  |
| 114 | Recipe with 3 herbs | *Moringa oleifera* Lam. | Leaves | Doesn't matter | Supply of essential nutrients Stimulates appetite Improves digestion Strengthens the immune system Fever Diarrhea | Infusion | On the recommendation of the herbalist or herbalist | Bamboo glass | 5.56 |
|  |  | *Elaeis guineensis* Jacq. | Bark | Whatever |  |  |  |  |  |
|  |  | *Pavetta crassipes* K.Schum. | Barks | Whatever |  |  |  |  |  |
| 115 | Recipe with 3 herbs | *Moringa oleifera* Lam. | Leaves | Whatever | Supply of essential nutrients Stimulation of appetite Improved digestion Fever | Infusion | On the recommendation of the herbalist or herbalist | Bamboo glass | 5.56 |
|  |  | *Sterculia setigera* Delile | Bark | Whatever |  |  |  |  |  |
|  |  | *Senna occidentalis* (L.) Link | Leaves | Whatever |  |  |  |  |  |
| 116 | Recipe with 3 herbs | *Moringa oleifera* Lam. | Leaves | Whatever | Supply of essential nutrients Stimulation of appetite Improved digestion Strengthened immune system Fever | Infusion | On the recommendation of the traditherapist or herbalist | Bamboo glass | 5.56 |
|  |  | *Carica papaya* L. | Leaves | Whatever |  |  |  |  |  |
|  |  | *Elaeis guineensis* Jacq. | Bark | Doesn't matter |  |  |  |  |  |
| 117 | Recipe with 3 herbs | *Setaria sphacelata* (Schumach.) Stapf & C.E.Hubb. ex Moss | Leaves Roots | Fresh | Supply of essential nutrients Stimulates appetite Improves digestion Fever Strengthens the immune system | Infusion | Age Sex | Bamboo glass | 5.56 |
|  |  | *Ocimum gratissimum* L. | Leaves | Fresh |  |  |  |  |  |
|  |  | *Paullinia pinnata* L. | Leaves | Doesn't matter. |  |  |  |  |  |
| 118 | Recipe with 4 herbs | *Senna occidentalis* (L.) Link | Leaves | Fresh | Supply of essential nutrients Stimulation of appetite Improved digestion Strengthened immune system Diarrhea | Infusion | On the recommendation of the traditherapist or herbalist | Bamboo glass | 50 |
|  |  | *Moringa oleifera* Lam. | Leaves | Any |  |  |  |  |  |
|  |  | *Sterculia setigera* Delile | Bark | Whatever |  |  |  |  |  |
|  |  | *Elaeis guineensis* Jacq. | Bark | Whatever |  |  |  |  |  |
| 119 | Recipe with 4 herbs | *Moringa oleifera* Lam. | Leaves | Whatever | Supply of essential nutrients Stimulation of appetite Improved digestion Fever | Infusion | On the recommendation of the therapist or herbalist | Bamboo glass | 25 |
|  |  | *Elaeis guineensis* Jacq. | Bark | It doesn't matter |  |  |  |  |  |
|  |  | *Terminalia leiocarpa* (DC.) Baill. | Leaves Bark | Whatever |  |  |  |  |  |
|  |  | *Senna occidentalis* (L.) Link | Leaves | Doesn't matter |  |  |  |  |  |
| 120 | Recipe with 4 herbs | *Senna siamea* (Lam.) H.S.Irwin & Barneby | Leaves Roots | Dryer | Appetite stimulation | Infusion | Child's condition | Bamboo glass | 25 |
|  |  | *Jatropha curcas* L. | Leaves | Dry |  |  |  |  |  |
|  |  | *Cocos nucifera* L. | Bark | Fresh |  |  |  |  |  |
|  |  | *Cymbopogon citratus* (DC.) Stapf | Leaves | Fresh Dry Any |  |  |  |  |  |
| 121 | Recipe with 5 herbs | *Pavetta crassipes* K.Schum. | Bark | Not important | Supply of essential nutrients Stimulation of appetite Improved digestion Strengthened immune system Fever Diarrhea | Infusion | On traditherapist's or herbalist's recommendation | Bamboo glass | 50 |
|  |  | *Elaeis guineensis* Jacq. | Bark | It doesn't matter |  |  |  |  |  |
|  |  | *Moringa oleifera* Lam. | Leaves | Doesn't matter |  |  |  |  |  |
|  |  | *Sterculia setigera* Delile | Bark | Doesn't matter |  |  |  |  |  |
|  |  | *Khaya senegalensis* (Desr.) A.Juss. | Barks | Doesn't matter |  |  |  |  |  |
| 122 | Recipe with 5 herbs | *Pavetta crassipes* K.Schum. | Leaves Barks | Whatever | Supply of essential nutrients Stimulation of appetite Improvement of digestion Strengthening of the immune system Fever Diarrhea | Infusion | On the recommendation of the herbalist or herbalist | Bamboo glass | 50 |
|  |  | *Moringa oleifera* Lam. | Leaves | Whatever |  |  |  |  |  |
|  |  | *Senna occidentalis* (L.) Link | Leaves | Fresh Whatever |  |  |  |  |  |
|  |  | *Elaeis guineensis* Jacq. | Bark | Whatever |  |  |  |  |  |
|  |  | *Sterculia setigera* Delile | Bark | Whatever |  |  |  |  |  |
