# Supplementary material for: Knowledge and practices of traditional management of child malnutrition and associated pathologies in Benin
Source: J Ethnobiol Ethnomed. 2024 May 2;20:47. doi: 10.1186/s13002-024-00684-x (PMC11064319; doi:10.1186/s13002-024-00684-x)
Supplement: Supplementary file 2 — Additional file 2: Table S8. Medicinal recipes used by traditional therapists for the traditional treatment of child malnutrition. [file 13002_2024_684_MOESM2_ESM.docx]

**Additional information**

**Table 8.** Medicinal recipes used by traditional healers for the traditional treatment of child malnutrition

| N° | Type of medicinal recipe | Scientific names of plants | Plant parts | State of use of the plant | What treatment does the recipe target? | Preparation method | Criteria for use | Posology | Citation frequency |
| --- | --- | --- | --- | --- | --- | --- | --- | --- | --- |
| 1 | **Monoplant recipe** | *Moringa oleifera* Lam. | Leaves Barks Roots Stems Seeds | Fresh, Whatever | Essential nutrients Digestion enhancement Immune system strengthening Fever Cough Diarrhea Convulsion Appetite stimulation and others | Decoction Powder Infusion Trituration Maceration | Child's condition Age | Coffee spoon Soup spoon Bamboo glass Tadokpemi glass and Others | 22.06 |
| 2 | **Monoplant recipe** | *Gymnanthemum amygdalinum* (Delile) Sch.Bip. | Seeds | It doesn't matter | Supply of essential nutrients Stimulation of appetite Improved digestion Strengthened immune system | Infusion | Child's condition Age |  | 16.18 |
| 3 | **Monoplant recipe** | *Pterocarpus erinaceus* Poir. | Barks Seeds | It doesn't matter | Supply of essential nutrients Improved digestion Strengthened immune system Fever Cough Diarrhea Appetite stimulation and others | Infusion Trituration Decoction Powder | Child's condition On the recommendation of the herbalist or traditherapist | Soup spoon Bamboo glass Coffee spoon | 8.82 |
| 4 | **Monoplant recipe** | *Momordica balsamina* L. | Stems Flowers Barks Leaves Roots Fruits Branches Seeds | Fresh, Whatever | Cough Diarrhea Fever Supply of essential nutrients Improved digestion Strengthened immune system Appetite stimulation Convulsion | Infusion Trituration Maceration Powder Decoction | Child's age Gender | Coffee spoon Soup spoon Bamboo glass Tadokpemi glass | 5.88 |
| 5 | **Monoplant recipe** | *Allium cepa* L. | Leaves | It doesn't matter | Supply of essential nutrients Immune system boost Cough Diarrhea Fever Improved digestion | Infusion PowderTrituration Maceration | Child's condition Age Sex On the recommendation of the herbalist or traditherapist | Coffee spoon Soup spoon | 4.41 |
| 6 | **Monoplant recipe** | *Carica papaya* L. | Roots Twigs Flowers Stems Barks Leaves Fruits Seeds | It doesn't matter | Supply of essential nutrients Appetite stimulation Other | Decoction Powder Trituration Maceration Infusion | Child's condition Age On the recommendation of the herbalist or traditherapist | Soup spoon Bamboo glass Tadokpemi glass Coffee spoon | 4.41 |
| 7 | **Monoplant recipe** | *Khaya senegalensis* (Desr.) A.Juss. | Leaves Barks | Fresh, Whatever | Supply of essential nutrients Stimulation of appetite Improvement of digestion Strengthening of the immune system Convulsion Cough Diarrhea Fever | Trituration, Powder, Infusion | Child's condition Age Sex | Coffee spoon, Tadokpemi glass and Others | 4.41 |
| 8 | **Monoplant recipe** | *Senna occidentalis* (L.) Link | Roots Twigs Flowers Stems Barks Leaves Fruits Seeds | It doesn't matter | Supply of essential nutrients Improvement of digestion Strengthening of the immune system Fever Cough Diarrhea | Infusion Trituration | Child's condition Age On recommendation of the traditherapist or herbalist | Coffee spoon Soup spoon Bamboo glass | 4.41 |
| 9 | **Monoplant recipe** | *Adansonia digitata* L. | Fruits Flowers Roots Leaves Barks Stems Branches Seeds | Doesn't matter | Stimulation of appetite Improvement of digestion Strengthening of the immune system, Supply of essential nutrients | Maceration Trituration Powder Decoction Infusion | On the recommendation of the traditherapist or herbalist, State of the child | Coffee spoon Soup spoon Bamboo glass Tadokpemi glass | 2.94 |
| 10 | **Monoplant recipe** | *Cassia sieberiana* DC. | Bark | Dry | Appetite stimulation Diarrhea Fever | Infusion Trituration | Child's condition Age Sex | Coffee spoon Soup spoon | 2.94 |
| 11 | **Monoplant recipe** | *Elaeis guineensis* Jacq. | Leaves | Fresh, Whatever | Supply of essential nutrients Fever Diarrhea Cough Stimulation of appetite Improvement of digestion | Decoction | Child's condition On the recommendation of the traditherapist or herbalist | Coffee spoon Bamboo glass Soup spoon | 2.94 |
| 12 | **Monoplant recipe** | *Ficus platyphylla* Delile | Leaves Bark | Doesn't matter | Fever Appetite stimulation | Infusion Trituration Powder | Child's condition Age Sex | Coffee spoon Soup spoon | 2.94 |
| 13 | **Monoplant recipe** | *Vachellia seyal* (Delile) P.J.H.Hurter | Leaves Flowers Fruits | It doesn't matter | Essential nutrients, Appetite stimulant, Immune system booster, Fever, Digestion enhancer Cough Diarrhea Other | Powder Decoction Trituration Infusion Maceration | Child's condition Age On the recommendation of the therapist or herbalist | Coffee spoon Soup spoon Bamboo glass | 2.94 |
| 14 | **Monoplant recipe** | *Citrus × aurantiifolia* (Christm.) Swingle | Leaves Barks | It doesn't matter | Essential nutrient intake Appetite stimulation Digestion enhancement Immune system strengthening Fever Cough Diarrhea | Infusion | Child's condition | Coffee spoon | 1.47 |
| 15 | **Monoplant recipe** | *Cola nitida* (Vent.) Schott & Endl | Leaves Stems Flowers | It does not matter | Supply of essential nutrients Fever Diarrhea Improved digestion | Infusion Maceration | Child's condition |  | 1.47 |
| 16 | **Monoplant recipe** | *Crateva adansonii* DC. | Leaves Barks Roots | It does not matter | Supply of essential nutrients Stimulation of appetite Improved digestion Fever Convulsion Cough | Infusion | Child's condition | Tadokpemi glass | 1.47 |
| 17 | **Monoplant recipe** | *Cymbopogon citratus* (DC.) Stapf | Leaves | Fresh | Supply of essential nutrients Stimulation of appetite Improvement of digestion Strengthening of the immune system | Infusion | Child's condition Age | Tadokpemi glass | 1.47 |
| .18 | **Monoplant recipe** | *Heliotropium indicum* L. | Leaves | Does not matter | Supply of essential nutrients Cough Diarrhea Fever Appetite stimulation | Decoction Powder Infusion | Child's condition On tradithérapeute's or herbalist's recommendation | Soup spoon Bamboo glass | 1.47 |
| 19 | **Monoplant recipe** | *Paullinia pinnata* L. | Leaves | Dry | Intake of essential nutrients Stimulation of appetite Improved digestion Strengthened immune system | Infusion Decoction Powder | Child's condition Age | Coffee spoon Soup spoon Bamboo glass | 1.47 |
| 20 | **Monoplant recipe** | *Piliostigma thonningii* (Schumach.) Milne-Redh. | Leaves | Fresh | Essential nutrients Fever Diarrhea Cough Appetite stimulation | Decoction | Child's condition | Soup spoon Bamboo glass | 1.47 |
| 21 | **Monoplant recipe** | *Senna siamea* (Lam.) H.S.Irwin & Barneby | Leaves Roots | Fresh | Intake of essential nutrients Stimulation of appetite Improvement of digestion Strengthening of the immune system | Infusion | Child's condition Age | Others | 1.47 |
| 22 | **Monoplant recipe** | *Spondias mombin* L. | Leaves | Fresh | Supply of essential nutrients Stimulation of appetite Improved digestion Strengthened immune system | Infusion | Child's condition Age | Others | 1.47 |
| 23 | **Monoplant recipe** | *Sterculia setigera* Delile | Barks | Fresh | Others | Maceration Trituration | Child's age Sex | Coffee spoon Soup spoon | 1.47 |
| 24 | **Recipe with two plants** | *Ficus platyphylla* Delile | Leaves Stems Barks | No matter | Stimulation of appetite Supply of essential nutrients Cough Diarrhea Fever | Infusion | Child's age Sex | Bamboo glass | 6.90 |
|  |  | *Moringa oleifera* Lam. | Leaves Barks Stems | Whatever |  |  |  |  |  |
| 25 | **Recipe with 2 plants** | *Moringa oleifera* Lam. | Leaves | Whatever | Supply of essential nutrients Fever Cough Diarrhea Stimulation of appetite Improved digestion Strengthened immune system | Decoction Powder | Child's condition | Coffee spoon Soup spoon Bamboo glass | 6.90 |
|  |  | *Senna occidentalis* (L.) Link | Leaves Roots Stems Seeds | Does not matter |  |  |  |  |  |
| 26 | **Recipe with 2 plants** | *Elaeis guineensis* Jacq. | Bark | It does not matter | Supply of essential nutrients Cough Diarrhea Fever Stimulation of appetite Improved digestion Strengthened immune system | Decoction | Child's condition On the recommendation of the traditherapist or herbalist | Coffee spoon Soup spoon Bamboo glass | 6.90 |
|  |  | *Moringa oleifera* Lam. | Leaves | Whatever |  |  |  |  |  |
| 27 | **Recipe with 2 plants** | *Elaeis guineensis* Jacq. | Barks | Whatever | Supply of essential nutrients Stimulation of appetite Improved digestion Strengthened immune system Fever | Infusion | On the recommendation of the traditherapist or herbalist | Bamboo glass | 6.90 |
|  |  | *Sterculia setigera* Delile | Leaves | Does not matter |  |  |  |  |  |
| 28 | **Recipe with 2 plants** | *Pavetta corymbosa* (DC.) F.N.Williams | Leaves | Dry | Supply of essential nutrients Stimulation of appetite Improved digestion Strengthened immune system | Powder Trituration | On the recommendation of the traditherapist or herbalist | Bamboo glass | 3.45 |
|  |  | *Senna italica* Mill. | Leaves | Fresh |  |  |  |  |  |
| 29 | **Recipe with two plants** | *Monodora myristica* (Gaertn.) Dunal | Fruit Stems | Doesn't matter | Improved digestion Diarrhea | Powder Decoction Infusion Trituration Maceration | Sex | Coffee spoon Soup spoon Tadokpemi glass | 3.45 |
|  |  | *Croton gratissimus* Burch. | leaf | Does not matter |  |  |  |  |  |
| 30 | **Recipe with 2 herbs** | *Garcinia kola* Heckel | Barks Roots Flowers | Does not matter | Supply of essential nutrients Improved digestion Fever | Maceration Trituration Powder Infusion Decoction | On traditherapist's or herbalist's recommendation Child's condition | Bamboo glass Coffee spoon Soup spoon Tadokpemi glass  . | 3.45 |
|  |  | *Newbouldia laevis* (P.Beauv.) Seem. ex Bureau | leaf |  |  |  |  |  |  |
| 31 | **Recipe with 2 herbs** | *Cola nitida* (Vent.) Schott & Endl | Flowers Stems Roots | Does not matter | Supply of essential nutrients Digestion improved Immune system strengthened Fever | Infusion Powder Trituration Maceration Decoction | Age Child's condition | Tadokpemi glass Soup spoon Bamboo glass Coffee spoon | 3.45 |
|  |  | *Mentha × piperita* L. | leaf | It doesn't matter |  |  |  |  |  |
| 32 | **Recipe with 2 herbs** | *Ocimum americanum* L*.* | Bark | Whatever | Immune system boost Convulsion | Maceration Trituration Decoction | On traditherapist's or herbalist's recommendation | Coffee spoon Soup spoon Bamboo glass Tadokpemi glass | 3.45 |
|  |  | *Xylopia aethiopica* (Dunal) A.Rich. | Stems Fruits | Whatever |  |  |  |  |  |
| 33 | **Recipe with 2 herbs** | *Zingiber officinale* Roscoe | Leaves | Whatever | Appetite stimulant Immune system booster | Maceration Trituration Decoction Powder Infusion | Sex On the recommendation of the herbalist or traditherapist Child's condition | Coffee spoon Soup spoon Bamboo glass Tadokpemi glass | 3.45 |
|  |  | *Xylopia aethiopica* (Dunal) A.Rich. | Roots Stems | Whatever |  |  |  |  |  |
| 34 | **Recipe with 2 herbs** | *Kalanchoe pinnata* (Lam.) Pers.. | Bark | Whatever | Strengthens the immune system Cough | Decoction Trituration Maceration | Age | Coffee spoon | 3.45 |
|  |  | *Xylopia aethiopica* (Dunal) A.Rich. | Stems Flowers | Whatever |  |  |  |  |  |
| 35 | **Recipe with 2 herbs** | *Calotropis procera* (Aiton) Dryand. | Barks Stems | Whatever | Essential nutrients Diarrhea Fever Improved digestion | Maceration Infusion | Child's condition | Coffee spoon Tadokpemi glass | 3.45 |
|  |  | *Xylopia aethiopica* (Dunal) A.Rich. | Flowers Fruits | Whatever |  |  |  |  |  |
| 36 | **Recipe with 2 herbs** | *Moringa oleifera* Lam. | Leaves | Whatever | Diarrhea Supply of essential nutrients Stimulation of appetite Strengthening of the immune system  . | Infusion Maceration | Child's condition Sex | Coffee spoon Tadokpemi glass Bamboo glass | 3.45 |
|  |  | *Crateva adansonii* DC. | Bark | Whatever |  |  |  |  |  |
| 37 | **Recipe with 2 herbs** | *Croton gratissimus* Burch. | Leaves | Whatever | Essential nutrients Appetite stimulation Cough Diarrhea | Infusion Maceration | Child's condition | Tadokpemi glass | 3.45 |
|  |  | *Calotropis procera* (Aiton) Dryand. | Leaves | Whatever |  |  |  |  |  |
| 38 | **Recipe with 2 herbs** | *Khaya senegalensis* (Desr.) A.Juss. | Leaves | Whatever | Supply of essential nutrients Appetite stimulation Cough Diarrhoea | Infusion Maceration | Child's condition | Tadokpemi glass | 3.45 |
|  |  | *Senna siamea* (Lam.) H.S.Irwin & Barneby | Bark | Whatever |  |  |  |  |  |
| 39 | **Recipe with 2 herbs** | *Mangifera indica* L*.* | Leaves | Whatever | Essential nutrients Diarrhea Fever Improved digestion | Maceration Infusion | Child's condition | Coffee spoon Tadokpemi glass | 3.45 |
|  |  | *Hibiscus cannabinus* L*.* | Leaves | Whatever |  |  |  |  |  |
| 40 | **Recipe with 2 herbs** | *Allium cepa* L. | Leaves | Whatever | Essential nutrients Appetite stimulation Fever Convulsion Cough Diarrhea | Decoction Infusion Powder Trituration | Child's condition On the recommendation of the traditherapist or herbalist | Soup spoon Bamboo glass | 3.45 |
|  |  | *Momordica balsamina* L. | Leaves Flowers Fruits | Doesn't matter |  |  |  |  |  |
| 41 | **Monoplant Recipe with 2 plants** | *Ficus platyphylla* Delile | Branches | Fresh | Supply of essential nutrients Appetite stimulation Cough Diarrhea | Infusion Maceration | Child's condition | Tadokpemi glass | 3.45 |
| 42 | **Recipe with 2 plants** | *Moringa oleifera* Lam. | Leaves | Doesn't matter | Supply of essential nutrients Stimulation of appetite Improved digestion Strengthened immune system Fever | Infusion | On the recommendation of the traditherapist or herbalist | Bamboo glass | 3.45 |
|  |  | *Pterocarpus erinaceus* Poir. | Bark | Does not matter |  |  |  |  |  |
| 43 | **Recipe with 2 plants** | *Ximenia americana* L. | Bark | Whatever | Supply of essential nutrients Stimulation of appetite Fever Diarrhea | Infusion | On the recommendation of the herbalist or herbalist | Bamboo glass | 3.45 |
|  |  | *Moringa oleifera* Lam. | Leaves | Doesn't matter |  |  |  |  |  |
| 44 | **Recipe with 2 plants** | *Senna occidentalis* (L.) Link | Leaves Roots Stems Seeds | It doesn't matter | Supply of essential nutrients Stimulation of appetite Fever Diarrhea | Infusion | On the recommendation of the traditherapist or herbalist | Bamboo glass | 3.45 |
|  |  | *Pterocarpus erinaceus* Poir. | Bark | Whatever |  |  |  |  |  |
| 45 | **Recipe with 2 plants** | *Moringa oleifera* Lam. | Leaves | Doesn't matter | Supply of essential nutrients Stimulation of appetite Fever Diarrhoea | Infusion | On the recommendation of the traditherapist or herbalist | Bamboo glass | 3.45 |
|  |  | *Allium cepa* L. | Leaves | Fresh |  |  |  |  |  |
| 46 | **Recipe with 2 plants** | *Momordica charantia* L*.* | Barks Roots Fruits Branches | Doesn't matter | Essential nutrients | Infusion Maceration Decoction Powder Trituration | On the recommendation of the traditherapist or herbalist | Soup spoon Bamboo glass Tadokpemi glass Coffee spoon | 3.45 |
|  |  | *Nauclea latifolia* Sm. | Flowers Stems Leaves |  |  |  |  |  |  |
| 47 | **Recipe with 2 plants** | *Adansonia digitata* L. | Leaves | Fresh | Supply of essential nutrients Improved digestion Strengthened immune system Fever | Infusion Powder Trituration Maceration Decoction | Age Condition of child | Tadokpemi glass Soup spoon Bamboo glass Coffee spoon | 3.45 |
|  |  | *Moringa oleifera* Lam. | Leaves | Doesn't matter |  |  |  |  |  |
| 48 | **Recipe with 2 plants** | *Moringa oleifera* Lam. | Leaves | Whatever | Supply of essential nutrients Stimulation of appetite Improvement of digestion | Infusion | On recommendation of the traditherapist or herbalist | Bamboo glass | 3.45 |
|  |  | *Elaeis guineensis* Jacq. | Bark | Whatever |  |  |  |  |  |
| 49 | **Recipe with 3 plants** | *Sterculia setigera* Delile | Barks | Whatever | Stimulation of appetite Improved digestion Fever Diarrhea | Infusion | On the recommendation of the traditherapist or herbalist | Bamboo glass | 9.09 |
|  |  | *Elaeis guineensis* Jacq. | Bark | It doesn't matter |  |  |  |  |  |
|  |  | *Moringa oleifera* Lam. | Bark Leaves | Whatever |  |  |  |  |  |
| 50 | **Recipe with 3 plants** | *Carica papaya* L. | Leaves | Whatever | Supply of essential nutrients Stimulation of appetite Fever Diarrhea | Infusion | On the recommendation of the traditherapist or herbalist | Bamboo glass | 9.09 |
|  |  | *Senna occidentalis* (L.) Link | Leaves | Whatever |  |  |  |  |  |
|  |  | *Moringa oleifera* Lam. | Leaves | Whatever |  |  |  |  |  |
| 51 | **Recipe with 3 plants** | *Terminalia leiocarpa* (DC.) Baill. | Leaves Barks | Whatever | Supply of essential nutrients Cough Diarrhea Fever | Decoction | Child's condition On traditherapist's or herbalist's recommendation | Coffee spoon Soup spoon Bamboo glass | 9.09 |
|  |  | *Moringa oleifera* Lam. | Leaves | Whatever |  |  |  |  |  |
|  |  | *Sterculia setigera* Delile |  | Whatever |  |  |  |  |  |
| 52 | **Recipe with 3 plants** | *Uvaria chamae* P.Beauv. | Bark | Whatever | Stimulation of appetite Strengthening of the immune system | Trituration Maceration | Age Sex | Coffee spoon Soup spoon Bamboo glass Tadokpemi glass | 4.55 |
|  |  | *Xylopia aethiopica* (Dunal) A.Rich. | Flowers Stems | Whatever |  |  |  |  |  |
|  |  | *Croton gratissimus* Burch. | Leaves Roots | Whatever |  |  |  |  |  |
| 53 | **Recipe with 3 plants** | *Jatropha curcas* L. | Leaves | Whatever | Diarrhea Stimulation of appetite Improved digestion Supply of essential nutrients | Infusion | Child's condition | Coffee spoon Tadokpemi glass | 4.55 |
|  |  | *Carica papaya* L. | Roots | Whatever |  |  |  |  |  |
|  |  | *Arachis hypogaea* L | Bark | Whatever |  |  |  |  |  |
| 54 | **Recipe with 3 plants** | *Arachis hypogaea* L | Leaves | Whatever | Supply of essential nutrients Stimulation of appetite Diarrhea Improved digestion | Infusion Maceration | Child's condition | Tadokpemi glass | 4.55 |
|  |  | *Hibiscus cannabinus* L*.* | Leaves | Whatever |  |  |  |  |  |
|  |  | *Crateva adansonii* DC. | Leaves | Whatever |  |  |  |  |  |
| 55 | **Recipe with 3 plants** | *Moringa oleifera* Lam. | Leaves | Whatever | Supply of essential nutrients Convulsion Diarrhea | Decoction Infusion Trituration Maceration | Child's condition | Tadokpemi glass | 4.55 |
|  |  | *Anonychium africanum* (Guill. & Perr.) C.E.Hughes & G.P.Lewis | Leaves | Whatever |  |  |  |  |  |
|  |  | *Mangifera indica* L. | Leaves | Whatever |  |  |  |  |  |
| 56 | **Recipe with 3 plants** | *Khaya senegalensis* (Desr.) A.Juss. | Leaves | Whatever | Essential nutrients Fever Appetite stimulation Digestion improvement | Infusion | Child's condition | Tadokpemi glass | 4.55 |
|  |  | *Phyllanthus amarus* Schumach. & Thonn. | Bark | Whatever |  |  |  |  |  |
|  |  | *Carica papaya* L. | Roots | Whatever |  |  |  |  |  |
| 57 | **Recipe with 3 plants** | *Carica papaya* L. | Leaves | Whatever | Supply of essential nutrients Stimulation of appetite Improved digestion Diarrhea Strengthening of the immune system | Infusion | Child's condition | Tadokpemi glass | 4.55 |
|  |  | *Khaya senegalensis* (Desr.) A.Juss. | Bark | Whatever |  |  |  |  |  |
|  |  | *Hibiscus cannabinus* L*.* |  | Whatever |  |  |  |  |  |
| 58 | **Recipe with 3 plants** | *Crateva adansonii* DC. | Leaves | Whatever | Supply of essential nutrients Stimulation of appetite Improved digestion Fever Convulsion Cough | Infusion | Child's condition | Glass tadokpemi | 4.55 |
|  |  | *Ocimum gratissimum* L. | Bark | Whatever |  |  |  |  |  |
|  |  | *Senna siamea* (Lam.) H.S.Irwin & Barneby | Roots | Whatever |  |  |  |  |  |
| 59 | **Recipe with 3 plants** | *Adansonia digitata* L. | Leaves | Fresh Whatever | Supply of essential nutrients Stimulation of appetite Improved digestion Fever Cough Diarrhea | Infusion Decoction Trituration | Child's condition Age On the recommendation of the traditherapist or herbalist | Coffee spoon Tadokpemi glass | 4.55 |
|  |  | *Ocimum gratissimum* L. | Leaves Roots | Fresh Whatever |  |  |  |  |  |
|  |  | *Moringa oleifera* Lam. | Roots | Doesn't matter |  |  |  |  |  |
| 60 | **Recipe with 3 plants** | *Croton gratissimus* Burch. | Leaves | Doesn't matter | Supply of essential nutrients Cough Diarrhea Improved digestion | Decoction Maceration | Child's condition | Coffee spoon Tadokpemi glass | 4.55 |
|  |  | *Phyllanthus amarus* Schumach. & Thonn. |  | Whatever |  |  |  |  |  |
|  |  | *Crateva adansonii* DC. |  | Doesn't matter |  |  |  |  |  |
| 61 | **Recipe with 3 plants** | *Moringa oleifera* Lam*.* | Leaves | Doesn't matter | Improved digestion | Infusion | Age Sex | Coffee spoon Soup spoon | 4.55 |
|  |  | *Senna occidentalis* (L.) Link | Leaves | Fraîche |  |  |  |  |  |
|  |  | *Elaeis guineensis* Jacq. | Bark | Doesn't matter |  |  |  |  |  |
| 62 | **Recipe with 3 plants** | *Moringa oleifera* Lam. | Leaves | Doesn't matter | Essential nutrients Appetite stimulation Diarrhea | Infusion Decoction Trituration Maceration | Child's condition | Tadokpemi glass | 4.55 |
|  |  | *Crateva adansonii* DC. | Leaves | Doesn't matter |  |  |  |  |  |
|  |  | *Psidium guajava* L. | Leaves | Doesn't matter |  |  |  |  |  |
| 63 | **Recipe with 3 plants** | *Ocimum gratissimum* L. | Leaves | Doesn't matter | Essential nutrients Appetite stimulating Diarrhea | Infusion Decoction Trituration Maceration | Child's condition | Tadokpemi glass | 4.55 |
|  |  | *Crateva adansonii* DC. | Roots | Doesn't matter |  |  |  |  |  |
|  |  | *Hibiscus cannabinus* L |  | Doesn't matter |  |  |  |  |  |
| 64 | **Recipe with 3 plants** | *Senna occidentalis* (L.) Link | Leaves | Doesn't matter | Supply of essential nutrients Appetite stimulation Diarrhoea | Infusion Decoction Trituration Maceration | Child's condition | Tadokpemi glass | 4.55 |
|  |  | *Sterculia setigera* Delile | Bark | Doesn't matter |  |  |  |  |  |
|  |  | *Moringa oleifera* Lam. | Bark | Doesn't matter |  |  |  |  |  |
| 65 | **Recipe with 3 plants** | *Senna occidentalis* (L.) Link | Leaves | Doesn't matter | Supply of essential nutrients Improved digestion Fever Diarrhea | Infusion | On the recommendation of the traditherapist or herbalist | Bamboo glass | 455 |
|  |  | *Sterculia setigera* Delile | Bark | Doesn't matter |  |  |  |  |  |
|  |  | *Moringa oleifera* Lam. | Bark | Doesn't matter |  |  |  |  |  |
| 66 | **Recipe with 3 plants** | *Moringa oleifera* Lam. | Leaves | Fresh | Improves digestion | Infusion | Age Sex | Coffee spoon Soup spoon | 4.55 |
|  |  | *Elaeis guineensis* Jacq. | Bark | Fresh Doesn't matter |  |  |  |  |  |
|  |  | *Sterculia setigera* Delile | Leaves | Doesn't matter |  |  |  |  |  |
| 67 | **Recipe with 3 plants** | *Elaeis guineensis* Jacq. | Bark | Doesn't matter | Supply of essential nutrients Stimulation of appetite Improved digestion Strengthened immune system Fever | Infusion | On traditherapist's or herbalist's recommendation | Bamboo glass | 4.55 |
|  |  | *Pavetta crassipes* K.Schum. | Bark | Doesn't matter |  |  |  |  |  |
|  |  | *Moringa oleifera* Lam. | Seeds | Doesn't matter |  |  |  |  |  |
| 68 | **Recipe with 4 plants** | *Calotropis procera* (Aiton) W.T.Aiton | Leaves | Doesn't matter | Supply of essential nutrients Stimulation of appetite Diarrhea Strengthening of the immune system | Infusion Maceration | Child's condition | Coffee spoon Tadokpemi glass | 33.33 |
|  |  | *Anonychium africanum* (Guill. & Perr.) C.E.Hughes & G.P.Lewis | Leaves Roots Barks | Doesn't matter |  |  |  |  |  |
|  |  | *Hibiscus cannabinus* L*.* | Roots | Doesn't matter |  |  |  |  |  |
|  |  | *Newbouldia laevis* (P.Beauv.) Seem. ex Bureau | Barks | Doesn't matter |  |  |  |  |  |
| 69 | **Recipe with 4 plants** | *Senna siamea* (Lam.) H.S.Irwin & Barneby | Leaves | Fresh Doesn't matter | Supply of essential nutrients Stimulation of appetite Improved digestion Convulsion Diarrhea | Infusion | Child's condition | Tadokpemi glass | 33.33 |
|  |  | *Chromolaena odorata* (L.) R.M.King & H.Rob | Bark | Fresh Doesn't matter |  |  |  |  |  |
|  |  | *Hibiscus cannabinus* L*.* |  | Fresh Doesn't matter |  |  |  |  |  |
|  |  | *Crateva adansonii* DC. |  | Fresh Doesn't matter |  |  |  |  |  |
| 70 | **Recipe with 4 plants** | *Hibiscus cannabinus* L*.* | Roots | Doesn't matter | Supply of essential nutrients Stimulation of appetite Improved digestion Fever Cough Diarrhea | Infusion Decoction Trituration | Child's condition Age On the recommendation of the traditherapist or herbalist | Coffee spoon Tadokpemi glass | 33.33 |
|  |  | *Senna siamea* (Lam.) H.S.Irwin & Barneby | Bark | Doesn't matter |  |  |  |  |  |
|  |  | *Moringa oleifera* Lam. | Leaves | Doesn't matter |  |  |  |  |  |
|  |  | *Khaya senegalensis* (Desr.) A.Juss. | Leaves | Doesn't matter |  |  |  |  |  |
| 71 | **Recipe with 5 plants** | *Senna siamea (Lam.) H.S. Irwin & Barneby.* | Barks Roots | Doesn't matter | Supply of essential nutrients Stimulation of appetite Improvement of digestion | Powder Infusion Maceration Trituration Decoction | Child's condition Age | Soup spoon Bamboo glass Tadokpemi glass Coffee spoon | 100 |
|  |  | *Hibiscus acetosella* Welw. ex Hiern | Flowers Fruits | Doesn't matter |  |  |  |  |  |
|  |  | *Newbouldia laevis* (P.Beauv.) Seem. ex Bureau | leaf | Doesn't matter |  |  |  |  |  |
|  |  | *Momordica charantia* L*.* | Leaves | Doesn't matter |  |  |  |  |  |
|  |  | *Carica papaya* L. | Leaves | Doesn't matter |  |  |  |  |  |
