# Supplementary material for: Knowledge and practices of traditional management of child malnutrition and associated pathologies in Benin
Source: J Ethnobiol Ethnomed. 2024 May 2;20:47. doi: 10.1186/s13002-024-00684-x (PMC11064319; doi:10.1186/s13002-024-00684-x)
Supplement: Supplementary file 3 — Additional file 3: Table S9. Data on medicinal plants used by informants. [file 13002_2024_684_MOESM3_ESM.docx]

**Additional information**

**Table 9.** Data on medicinal plants used by informants

|  | | |  | | | | Mothers | | | | | Traditional healers | | |  | |  | | |
| --- | --- | --- | --- | --- | --- | --- | --- | --- | --- | --- | --- | --- | --- | --- | --- | --- | --- | --- | --- |
| N° | **Vouchers number** | **Medicinal plants** | | **Vernacular names (Fon, Goun, Adja, Sahouè, Yoruba)** | **Botanical families** | **Plant parts** | | **Fc (%)** | **Cpr (%)** | **IF (%)** | | **Fc (%)** | **Cpr (%)** | **IF (%)** | **TFc** | **Traditional uses** | |  |  |
| 1 | YH 882/HNB | *Moringa oleifera* Lam. | | Kpanouman | Moringaceae | Leaves | | 25.42 | 10.66 | 29.73 | | 19.91 | 30.99 | 21 | 45.33 | The leaves were used in paralysis, fever, wound, cough, enlarged liver and spleen | |  |  |
| 2 | YH 910/HNB | *Senna siamea* (Lam.) H.S.Irwin & Barneby | | Zangalama | Fabaceae | Leaves | | 6.59 | 4.92 | 7.71 | | 2.37 | 5.63 | 0.5 | 8.96 | Leaves’s were used locally as antimalarial medications | |  |  |
| 3 | YH 890/HNB | *Carica papaya* L. | | Kpinman | Caricaceae | Leaves | | 6 | 5.74 | | 7.01 | 3.79 | 8.45 | 4 | 9.79 | The leaves are traditionally used to treat various illnesses, such as dengue, fever, asthma, colic, beriberi, digestive disorders, hypertension, arthritis, ringworm and jaundice. They have healing, anticancer, hypolipidemic and hypoglycemic properties. | | |  |
| 4 | YH 885/HNB | *Phyllanthus amarus* Schumach. & Thonn. | | Hlinwe | Phyllanthaceae | Leaves | | 6 | 0.82 | 7.01 | | 0.95 | 2.82 | 1 | 6.95 | The leaves were used in the problems of stomach, genitourinary system, liver, kidey and spleen | |  |  |
| 5 | YH 920/HNB | *Ocimum gratissimum* L. | | Tchayo | Lamiaceae | Leaves | | 5.04 | 3.28 | 5.89 | | 1.42 | 4.23 | 1.5 | 6.46 | The leaves is used in the treatment of various diseases such as diabetes, cancer, inflammation, anaemia, diarrhoea, pains, fungal and bacterial infections. | |  |  |
| 6 | YH 927/HNB | *Adansonia digitata* L. | | Baobaob | Malvaceae | Leaves Fruits | | 4.08 | 0.82 | 4.77 | | 1.9 | 4.23 | 2 | 5.98 | Its fruist pulp are very rich in vitamin C and can be used as a seasoning, an aperitif and to make juice.  The leaves have a higher nutritional quality and contain significant levels of vitamin A | |  |  |
| 7 | YH 941/HNB | *Khaya senegalensis* (Desr.) A.Juss. | | Zounza | Meliaceae | Barks | | 3.84 | 4.1 | 4.49 | | 3.32 | 7.04 | 3.5 | 7.16 | They are used to treat deworming, malaria, fever, jaundice, colic, scabies, leprosy, anemia diarrhea, migraine and gastrointestinal diseases. | |  |  |
| 8 | YH 901/HNB | *Vitellaria paradoxa* C.F.Gaertn. | | Sinhui | Sapotaceae | Leaves | | 3.6 | 5.74 | 4.21 | |  |  |  | 3.6 | The leaves are used to treat various illnesses and injuries including stomach aches, headaches, fever, jaundice, etc. | |  |  |
| 9 | YH 891/HNB | *Senna occidentalis* (L.) Link | | Kinkeriba | Fabaceae | Leaves Barks | | 3.36 | 10.66 | 3.93 | | 5.21 | 8.45 | 5.5 | 8.57 | Leaves and barks are used as laxatives and purgatives. They are used to treat fever typhoid, malaria and hepatitis. | |  |  |
| 10 | YH 880/HNB | *Gymnanthemum amygdalinum* (Delile) Sch.Bip. | | Amavive | Asteraceae | Leaves | | 3.24 | 1.64 | 3.79 | | 7.58 | 1.41 | 8 | 10.82 | The leaves are used as an appetite stimulant and as a vegetable in stews and soups. They are also used in the indigenous treatment and management of several diseases such as malaria, diabeties, etc | |  |  |
| 11 | YH 912/HNB | *Elaeis guineensis* Jacq. | | Sêdé | Arecaceae | Branches Fruits | | 2.76 | 11.48 | 3.23 | | 5.69 | 11.27 | 6 | 8.45 | The fruits are used to make palm nut soup. The fruit mesocarp oil and palm kernel oil are administered as a poison antidote and used externally with several other herbs as a lotion to treat skin diseases. | |  |  |
| 12 | YH 954/HNB | *Sterculia setigera* Delile | | Takoundako | Malvaceae | Barks | | 2.16 | 9.84 | 2.52 | | 4.74 | 9.86 | 5 | 6.9 | The bark is used to treat cough, hypertension, headache, asthma, bronchitis, wounds, fe- ver, toothache, abscess, diarrhea, and snakebite | |  |  |
| 13 | YH 923/HNB | *Anonychium africanum* (Guill. & Perr.) C.E.Hughes & G.P.Lewis | | Kakè | Fabaceae | Leaves Barks Roots | | 1.92 | 2.46 | 2.24 | | 0.95 | 2.82 | 1 | 2.87 | The leaves in particular are used for the treatment of headache and toothache as well as various other head ailments. The roots are a diuretic and are used to treat gonorrhea, tooth and stomach-ache, dysentery and bronchitis. The bark is also used for the purpose of wound healing. | |  |  |
| 14 | YH 881/HNB | *Citrus × aurantiifolia* (Christm.) Swingle | | Klé man | Rutaceae | Leaves | | 1.68 | 2.46 | 1.96 | | 0.47 | 1.41 | 0.5 | 2.15 | The leaves are used for the treatment of wide panel of diseases like  stomach ache, vomiting, blood pressure, cough, cold,  bronchitis, ear ache, dysentery, diarrhea, abdominal pain  and fever. | |  |  |
| 15 | YH 936/HNB | *Pavetta crassipes* K.Schum. | | Dakplassou | Rubiaceae | Barks, Leaves | | 1.68 | 8.2 | 1.96 | | 0.47 | 1.41 | 0.5 | 2.15 | The leaves are used in the treatment of liver necrosis hypertension, diabetes and diarrhea, the treatment of malaria, infections diseases, and sexual asthenia. The barks are used in skin disorders, fever, abdominal pain,  arthritis, stomach disorders  . | |  |  |
| 16 | YH 903/HNB | *Momordica charantia* L*.* | | Yinsinkin | Cucurbitaceae | Stems Leaves | | 1.56 | 1.64 | 1.82 | | 1.9 | 2.82 | 2 | 3.46 | The leaves are used to cure several diseases like: gout, rheumatism, colic, worms, illness  of liver and spleen. The stems are as a hypolipidemic agent. | |  |  |
| 17 | YH 889/HNB | *Mangifera indica* L. | | Amangan | Anacardiaceae | Leaves Roots | | 1.44 | 1.64 | 1.68 | | 0.95 | 2.82 | 1 | 2.39 | The leaves produce a cooling effect and are used to treated fever and colds.  The root are used  to treat abscesses, broken horn,  diarrhea, indigestion, bacillosis, bloody dysentery,  excessive urination, tetanus. | |  |  |
| 18 | YH 948/HNB | *Paullinia pinnata* L. | | Goudouma gbadama cléema+kion | Sapindaceae | Leaves Roots | | 1.44 | 3.28 | 1.68 | | 0.47 | 1.41 | 0.5 | 1.91 | The leaves and roots are used to treat several diseases including rheumatism, weakness, impotence, ulcers, haemorrhoids, wounds, malaria, fever, hypertension, contraceptive, syphilis, convulsion, typhoid, and diarrhea. | |  |  |
| 19 | YH 917/HNB | *Cymbopogon citratus* (DC.) Stapf | | Tiigbé , Timatisê | Poaceae | Leaves | | 1.2 | 2.46 | 1.4 | | 0.47 | 1.41 | 0.5 | 1.67 | The leaves have been traditionally used as tea or decoction. There are also used to improve circulation, stabilise menstrual. | |  |  |
| 20 | YH 902/HNB | *Allium cepa* L. | | Touma | Amaryllidaceae | Leaves | | 0.96 | 1.64 | 1.12 | | 2.37 | 4.23 | 2.5 | 3.33 | The leaves contain a number of vitamins  selenium, and potassium and can can cure diabetes  mellitus, CVDs, and stomach cancer | |  |  |
| 21 | YH 937/HNB | *Glycine max* (L.) Merr. | | Soja | Fabaceae | Fruits Seeds | | 0.84 | 2.46 | 0.98 | |  |  |  | 0.84 | The seed and fruit are considered to be specific for the healthy functioning of bowels, heart, kidney, liver and stomach | |  |  |
| 22 | YH 952/HNB | *Pterocarpus erinaceus* Poir. | | Tolo-banda | Fabaceae | Barks | | 0.84 | 2.46 | 0.98 | |  |  |  | 0.84 | Barks are used  to treat inflammatory diseases such as inflammation, ulcer, rheumatism, and fever. | |  |  |
| 23 | YH 878/HNB | *Spondias mombin* L. | | Akikon | Anacardiaceae | Leaves | | 0.84 | 2.46 | 0.98 | | 0.47 | 1.41 | 0.5 | 1.31 | The home-made preparations (teas) of the leaves are utilized for treating diarrhea, emesis, hemorrhoids and throat inflammation. | |  |  |
| 24 | YH 929/HNB | *Arachis hypogaea* L. | | Azifloman | Fabaceae | Leaves Barks Roots | | 0.72 | 0.82 | 0.84 | | 0.47 | 2.82 | 1 | 1.19 | The leaves and root are used for the treatment of insomnia and inflammation. Roots are also used to treat prostate enlargement. Bark prevents malaria. | |  |  |
| 25 | YH 926/HNB | *Musa × paradisiaca* L. | | Kouékoué aloga | Musaceae | Leaves | | 0.72 | 1.64 | 0.84 | |  |  |  | 0.72 | The leaves are used to enhance endurance, heart health, the immune system, help induce sleep and for the treatment of diarrhoea. | |  |  |
| 26 | YH 906/HNB | *Cocos nucifera* L. | | Coco | Arecaceae | Fruits | | 0.6 | 2.46 | 0.7 | |  |  |  | 0.6 | The fruits can be eaten as food or used as medicine. They are used to treat fever and malaria. | |  |  |
| 27 | YH 898/HNB | *Croton gratissimus* Burch. | | Adjleleman | Euphorbiaceae | Leaves | | 0.6 | 0.82 | 0.7 | | 1.42 | 5.63 | 1.5 | 2.02 | The leaf decoction is used as anti-hypertensive, anti-microbial (urinary infections) and to treat malaria-linked fever. | |  |  |
| 28 | YH 934/HNB | *Erythrina senegalensis* DC. | | Kpaklessiman | Fabaceae | Leaves | | 0.6 | 0.82 | 0.7 | |  |  |  | 0.6 | The leaves are used to treat malaria, gastrointestinal disorders, fever, dizziness, secondary sterility, diarrhea, jaundice, nose bleeding and relieve pain. | |  |  |
| 29 | YH 949/HNB | *Pavetta corymbosa* (DC.) F.N.Williams | | Lohoui gnigloé gbeton djélélé | Rubiaceae | Leaves | | 0.6 | 0.82 | 0.7 | | 0.47 | 1.41 | 0.5 | 1.07 | The leaves are used in the treatment of malaria. | |  |  |
| 30 | YH 951/HNB | *Psidium guajava* L. | | Kinkoun ma /Leaves de goyavier ; yinglema /Leaves de corosol | Myrtaceae | Leaves | | 0.6 | 0.82 | 0.7 | | 0.47 | 1.41 | 0.5 | 1.07 | The leaves are used in the treatment of diseases such as diarrhea, rheumatism, and diabetes. | |  |  |
| 31 | YH 955/HNB | *Terminalia leiocarpa* (DC.) Baill. | | gonga banda | Combretaceae | Ecorce | | 0.48 | 1.64 | 0.56 | | 0.95 | 1.41 | 1 | 1.43 | The bark is used against diarrhoea and dysentery. | |  |  |
| 32 | YH 888/HNB | *Calotropis procera* (Aiton) Dryand. | | Amonman, kpintoman | Apocynaceae | Leaves | | 0.48 | 0.82 | 0.56 | | 1.42 | 4.23 | 1.5 | 1.9 | The leaf of Calotropis procera contains an enzyme  called calotropain, which induces cow or goat milk  coagulation. | |  |  |
| 33 | YH 907/HNB | *Solanum macrocarpon* L. | | Gboma | Solanaceae | Leaves | | 0.48 | 0.82 | 0.56 | |  |  |  | 0.48 | The leaves can be boiled to extract the juice which can be used to alleviate jaundice, asthma, whooping cough, dyslipidemia, and diabetes. | |  |  |
| 34 | YH 918/HNB | *Crateva adansonii* DC. | | Hontonzouzin | Capparaceae | Leaves Barks | | 0.36 | 1.64 | 0.42 | | 3.79 | 11.27 | 4 | 4.15 | The leaves and bark are used to treat joint discomfort, ear and toothaches, eye infection, asthma, rheumatoid arthritis, and epilepsy. | |  |  |
| 35 | YH 919/HNB | *Mesosphaerum suaveolens* (L.) Kuntze | | Zansoukpeman | Lamiaceae | Leaves | | 0.36 | 0.82 | 0.42 | |  |  |  | 0.36 | The leaves of the plant are accepted as substituent infusion tea. which renowned for its effectiveness against benign painful attacks. | |  |  |
| 36 | YH 942/HNB | *Landolphia dulcis* (Sabine ex G.Don) Pichon | | Kombé | Apocynaceae | Fruits | | 0.36 | 1.64 | 0.42 | |  |  |  | 0.36 | The fruits are used in treatment of gastric ulcers, stomach cramps and vermifuge, gastrointestinal disorders, typhoid fever, bacterial etiology, constipation, diarrhea and food poisonings. | |  |  |
| 37 | YH 956/HNB | *Vachellia seyal* (Delile) P.J.H.Hurter | | Chaman | Fabaceae | Leaves | | 0.24 | 0.82 | 0.28 | | 0.95 | 1.41 | 1 | 1.19 | The leaves are used to treat allergy, inflammatory reactions. | |  |  |
| 38 | YH 925/HNB | *Aloe buettneri* A.Berger | | Mimian | Asphodelaceae | Leaves | | 0.24 | 0.82 | 0.28 | |  |  |  | 0.24 | The leaves are used to treat skin problems (burns, wounds, and anti-inflammatory processes) | |  |  |
| 39 | YH 928/HNB | *Annona senegalensis* Pers. | | Huinglo | Annonaceae | Leaves Fruits | | 0.24 | 0.82 | 0.28 | |  |  |  | 0.24 | The leaves have been used in treating yellow fever, tuberculosis, and small pox. The fruits are used as antidotes for venomous bites and in the management of diabetes and malaria. | |  |  |
| 40 | YH 932/HNB | *Combretum collinum* Fresen. | | Dassi | Combretaceae | Seeds | | 0.24 | 1.64 | 0.28 | |  |  |  | 0.24 | The seeds are used against inflammation, infections, diabetes, malaria, bleeding, diarrhea and digestive disorders and others as a diuretic. | |  |  |
| 41 | YH 909/HNB | *Heliotropium indicum* L. | | Kokolosoudinkpatche | Boraginaceae | Leaves | | 0.24 | 1.64 | 0.28 | | 0.47 | 1.41 | 0.5 | 0.71 | The leaf juice is used to treat the stings and boils of scorpions and insect bites and allergy. | |  |  |
| 42 | YH 908/HNB | *Hibiscus sabdariffa* L. | | Bissap | Malvaceae | Leaves | | 0.24 | 0.82 | 0.28 | |  |  |  | 0.24 | The leaves are used to prepare herbal drinks, wine, ice cream, flavouring agents, puddings, and cakes. They calm the contractions of the uterus, stomach, and intestines, remove its pain. | |  |  |
| 43 | YH 913/HNB | *Launaea taraxacifolia* (Willd.) Amin ex C.Jeffrey | | Wonto | Asteraceae | Leaves | | 0.24 | 0.82 | 0.28 | |  |  |  | 0.24 | The leaves are **eaten either fresh as a salad or cooked as a sauce.** | |  |  |
| 44 | YH 895/HNB | *Ocimum americanum* L. | | Hêviosso késsoukéssou | Lamiaceae | Leaves Roots | | 0.24 | 0.82 | 0.28 | | 0.47 | 1.41 | 0.5 | 0.71 | The leaves are used to take eye care and the root to trat malaria and fever. | |  |  |
| 45 | YH 946/HNB | *Oxytenanthera abyssinica* (A.Rich.) Munro | | Dawé | Poaceae | Leaves | | 0.24 | 0.82 | 0.28 | |  |  |  | 0.24 | The leaves are used for treatment of urinary problems and diabetes. | |  |  |
| 46 | YH 950/HNB | *Piliostigma thonningii* (Schumach.) Milne-Redh. | | Klonman | Fabaceae | Leaves | | 0.24 | 1.64 | 0.28 | | 0.47 | 1.41 | 0.5 | 0.71 | The decoction of the leaves is used for the treatment of ulcers, wounds, heart pain, arthritis, malaria, pyrexia, leprosy, sore throat, diarrhea, toothache, gingivitis, cough, and bronchitis. | |  |  |
| 47 | YH 915/HNB | *Ageratum conyzoides* L. | | Mi -ma ;chimadidé | Asteraceae | Leaves | | 0.12 | 0.82 | 0.14 | |  |  |  | 0.12 | The leaves crushed in water are applied intravaginally for uterine troubles and also given as emetic. They are applied to cuts, burns and sores (styptic) and externally for body rash. | |  |  |
| 48 | YH 879/HNB | *Azadirachta indica* A.Juss. | | Glinima(nime) | Meliaceae | Leaves | | 0.12 | 0.82 | 0.14 | |  |  |  | 0.12 | The leaves are used to treat dental and gastrointestinal disorders, malaria fevers, skin diseases. | |  |  |
| 49 | YH 896/HNB | *Cassytha filiformis* L. | | Aboéboéman | Lauraceae | Whole plant | | 0.12 | 0.82 | 0.14 | |  |  |  | 0.12 | It is considered astringent and diuretic and contains several aporphine alkaloids and is often used in African folk medicine to treat cancer, African trypanosomiasis and other diseases. | |  |  |
| 50 | YH 930/HNB | *Rhodognaphalon brevicuspe* (Sprague) Roberty | | Bantan | Malvaceae | Leaves | | 0.12 | 0.82 | 0.14 | |  |  |  | 0.12 | The leaves are used to treat fever, cough, hoarseness, and venereal diseases. | |  |  |
| 51 | YH 914/HNB | *Sesamum radiatum* Thonn. ex Hornem. | | Agbɔ | Pedaliaceae | Leaves | | 0.12 | 0.82 | 0.14 | |  |  |  | 0.12 | The leaves are used as vegetable and in treating some diseases. | |  |  |
| 52 | YH 893/HNB | *Citrus maxima* (Burm.) Merr. | | Paplemoussou | Rutaceae | Fruits | | 0.12 | 0.82 | 0.14 | |  |  |  | 0.12 | The fruits are used as **a** treatment of cough, swelling, epilepsy as well as beautification purposes. | |  |  |
| 53 | YH 931/HNB | *Cleistopholis patens* (Benth.) Engl. & Diels | | A goun touba | Annonaceae | Leaves | | 0.12 | 0.82 | 0.14 | |  |  |  | 0.12 | The leaves are used for the treatment of typhoid fever and urogenital infections. | |  |  |
| 54 | YH 933/HNB | *Ehretia cymosa* Thonn. | | Zoman | Boraginaceae | Leaves | | 0.12 | 0.82 | 0.14 | |  |  |  | 0.12 | They are used for the treatment of measles, diarrhea, epilepsy, convulsions, spasm. | |  |  |
| 55 | YH 892/HNB | *Euphorbia hirta* L. | | Anonsinma | Euphorbiaceae | Leaves | | 0.12 | 0.82 | 0.14 | |  |  |  | 0.12 | The leaves are used to treatment of ulcers, overcoming dysentery, digestive tract infections, and skin infections. | |  |  |
| 56 | YH 935/HNB | *Ficus platyphylla* Delile | | Kobé | Moraceae | Leaves Barks Stems | | 0.12 | 0.82 | 0.14 | | 2.37 | 4.23 | 2.5 | 2.49 | The stems and barks are **used** to treat malaria in Africa and in treating tuberculosis. The leaves **are used** to control overweight and obesity. | |  |  |
| 57 | YH 939/HNB | *Hibiscus cannabinus* L*.* | | Ko kossou | Malvaceae | Leaves | | 0.12 | 0.82 | 0.14 | | 3.32 | 9.86 | 3.5 | 3.44 | The leaves are used to treat various disorders, such as of the blood, diabetes, bilious, the throat, and coughs. | |  |  |
| 58 | YH 905/HNB | *Hyphaene thebaica* (L.) Mart*.* | | Kongou | Arecaceae | Seeds | | 0.12 | 0.82 | 0.14 | |  |  |  | 0.12 | The seed is used to treat sore eyes. | |  |  |
| 59 | YH 940/HNB | *Senna italica* Mill. | | Tikpa tikpa | Fabaceae | Leaves | | 0.12 | 0.82 | 0.14 | | 0.47 | 1.41 | 0.5 | 0.59 | The leaves are used in the treatment of diarrhoea. | |  |  |
| 60 | YH 916/HNB | *Irvingia gabonensis* (Aubry-Lecomte ex O'Rorke) Baill. | | Asolor ma ,mangagodonou | Fabaceae | Leaves Barks | | 0.12 | 0.82 | 0.14 | |  |  |  | 0.12 | The bark is used to treat dysentery, scabies, toothache, and skin diseases. In combination with palm oil, the leaves are used to stop hemorrhage in pregnant women. | |  |  |
| 61 | YH 921/HNB | *Jatropha curcas* L. | | gbaguidikpotĩn | Euphorbiaceae | Leaves | | 0.12 | 0.82 | 0.14 | | 0.47 | 1.41 | 0.5 | 0.59 | They are used to treat bacterial and fungal infections or febrile diseases, muscle pain or jaundice. | |  |  |
| 62 | YH 943/HNB | *Macrosphyra longistyla* (DC.) Hook.f. ex Hiern | | Ziguidiboé | Rubiaceae. | Barks | | 0.12 | 0.82 | 0.14 | |  |  |  | 0.12 | The barks are used to treat headache and fever. | |  |  |
| 63 | YH 887/HNB | *Manihot esculenta* Crantz | | Koutema (Leaves de manioc) | Euphorbiaceae | Leaves | | 0.12 | 0.82 | 0.14 | |  |  |  | 0.12 | They are used to treat hypertension, headache, and pain. | |  |  |
| 64 | YH 903/HNB | *Momordica balsamina* L. | | Badoma | Cucurbitaceae | Leaves | | 0.12 | 0.82 | 0.14 | | 1.9 | 2.82 | 2 | 2.02 | They are used to treat malaria. | |  |  |
| 65 | YH 894/HNB | *Newbouldia laevis* (P.Beauv.) Seem. ex Bureau | | Desigueman | Bignoniaceae | Leaves | | 0.12 | 0.82 | 0.14 | | 1.42 | 2.82 | 1.5 | 1.54 | The leaves are used for pain, inflammation, convulsion, and epilepsy. | |  |  |
| 66 | YH 947/HNB | *Parinari curatellifolia* Planch. ex Benth. | | Sougue | Chrysobalanaceae | Leaves Barks | | 0.12 | 0.82 | 0.14 | |  |  |  | 0.12 | The leaves and a decoction of the bark is used in the treatment of anaemia, pneumonia, abdominal complaints, and toothache. | |  |  |
| 67 | YH 953/HNB | *Setaria sphacelata* (Schumach.) Stapf & C.E.Hubb. ex Moss | | Gbalama ,chiayoé ma oquion goudouman | Poaceae | Leaves Roots | | 0.12 | 0.82 | 0.14 | |  |  |  | 0.12 | There are used as an external application in the treatment ofailment and as a food | |  |  |
| 68 | YH 883/HNB | *Anacardium occidentale* L. | | Acajou | Anacardiaceae | Barks | | 0.12 | 0.82 | 0.14 | |  |  |  | 0.12 | A decoction of the **bark** has been **used** as an antipyretic (fever). | |  |  |
| 69 | YH 952/HNB | *Pterocarpus erinaceus* Poir. | | Fabaceae | Tolo-banda | Barks | |  |  |  | | 3.79 | 4.23 | 4 | 3.79 | The bark is used to treat inflammation. | |  |  |
| 70 | YH 900/HNB | *Xylopia aethiopica* (Dunal) A.Rich. | | Annonaceae | kpedjedekoun | Flowers Fruits | |  |  |  | | 2.37 | 7.04 | 2.5 | 2.37 | They are usedas a spyce (food) and a carminative, purgative and revulsive to counter pain. | |  |  |
| 71 | YH 884/HNB | *Cassia sieberiana* DC. | | Fabaceae | sigandi | Barks | |  |  |  | | 0.95 | 1.41 | 1 | 0.95 | The barks are used for the treatment of inflammatory conditions, tiredness and joint pains. | |  |  |
| 72 | YH 922/HNB | *Cola nitida* (Vent.) Schott & Endl | | Malvaceae | Cola | Leaves Stems Flowers | |  |  |  | | 0.95 | 2.82 | 1 | 0.95 | They are used in the treatment of dysentery, diarrhoea, coughs, vomiting, and chest complaints. | |  |  |
| 73 | YH 897/HNB | *Kalanchoe pinnata* (Lam.) Pers. | | Crassulaceae | Aké-man | Barks | |  |  |  | | 0.47 | 1.41 | 0.5 | 0.47 | The bark is used in a decoction for stomach ache, thrush, colds, coughs, sore throat and asthma. | |  |  |
| 74 | YH 886/HNB | *Garcinia kola* Heckel | | Clusiaceae | Colas | Barks Roots Flowers | |  |  |  | | 0.47 | 1.41 | 0.5 | 0.47 | They are used to treat diarrhoea, dysentery kidneys diseases, and as a diuretic. | |  |  |
| 75 | YH 938/HNB | *Hibiscus acetosella* Welw. ex Hiern | | Malvaceae | guissin | Flowers Fruits | |  |  |  | | 0.47 | 1.41 | 0.5 | 0.47 | Flowers and fruits are used to make teas or other drinks where they contribute color rather than taste. | |  |  |
| 76 | YH 944/HNB | *Mentha × piperita* L. | | Lamiaceae | Manth | Leaves | |  |  |  | | 0.47 | 1.41 | 0.5 | 0.47 | The leaves are typically used fresh as a culinary herb and as a tea.They are also used as diuretic agent, used to treat cold, asthma. | |  |  |
| 77 | YH 945/HNB | *Monodora myristica* (Gaertn.) Dunal | | Annonaceae | Sasalikoun | Fruits Stems | |  |  |  | | 0.47 | 1.41 | 0.5 | 0.47 | The fruits are dried to be used in stews, soups, cakes and desserts. The stem bark is used in the treatments of hemorrhoids, stomach ache. | |  |  |
| 78 | YH 877/HNB | *Chromolaena odorata* (L.) R.M.King & H.Rob. | | Rubiaceae | agatou | Barks | |  |  |  | | 0.47 | 1.41 | 0.5 | 0.47 | Decoction of leaves and bark is used against diabetes. | |  |  |
| 79 | YH 904/HNB | *Nauclea latifolia* Sm. | | Rubiaceae | kodor | Flowers Stems Leaves | |  |  |  | | 0.47 | 1.41 | 0.5 | 0.47 | The stem, flowers and leaves are used for the treatment of malaria, stomach ache, fever, diarrhea. | |  |  |
| 80 | YH 924/HNB | *Uvaria chamae* P.Beauv. | | Annonaceae | Ayadaxa sin dor | Barks | |  |  |  | | 0.47 | 1.41 | 0.5 | 0.47 | They are used to treat gastroenteritis, vomiting, diarrhea, dysentery, wounds, sore throats, inflamed gums and a number of other ailments. | |  |  |
| 81 | YH 957/HNB | *Ximenia americana* L. | | Olacaceae | Tonga banda | Barks | |  |  |  | | 0.47 | 1.41 | 0.5 | 0.47 | They are used for treatment of different human ailments such as fevers, constipation, leprosy, infections of the eyes and ears. | |  |  |
| 82 | YH 899/HNB | *Zingiber officinale* Roscoe | | Zingiberaceae | Dotè | Leaves | |  |  |  | | 0.47 | 1.41 | 0.5 | 0.47 | They are used for food-flavouring. Also used for arthritis, cramps, sprains, sore throats, rheumatism, muscular aches, pains, vomiting, constipation, indigestion, hypertension, dementia, fever and infectious diseases. | |  |  |

**Legend:** Fc: Frequency of citation, Cpr: Contribution to recipes , IF: Indice of fidelity, TFc: Total Frequency of citation
